# Isolation and Characterisation of Alkaloids from Marine-Derived *Aspergillus fumigatus* SYPHU504 with Antiproliferative Activity

**DOI:** 10.3390/md24070247

**Published:** 2026-07-16

**Authors:** Xuelei Zhang, Yingshu Yu, Kai Liu, Yonghong Liu, Hong Zhang, Jiao Xiao

**Affiliations:** 1 Wuya College of Innovation, Shenyang Pharmaceutical University, Shenyang 110016, China; 15248089084@163.com (X.Z.); yuyingshu98@163.com (Y.Y.); 2 Guangxi Key Laboratory of Marine Drugs and Institute of Marine Drugs, Guangxi University of Chinese Medicine, Nanning 530200, China; kailiu@gxtcmu.edu.cn; 3 School of Clinical Pharmacy, Shenyang Pharmaceutical University, Shenyang 110016, China; 4 School of Traditional Chinese Materia Medica, Shenyang Pharmaceutical University, Shenyang 110016, China

**Keywords:** marine-derived fungi, secondary metabolites, alkaloids, anti-leukaemia

## Abstract

Four novel alkaloids, including three *γ*-lactam alkaloids (**1**–**3**) and one diketopiperazine (**4**), along with eight previously known compounds (**5**–**12**), were isolated from the marine-derived fungus *Aspergillus fumigatus* SYPHU504. Their structures, including the tentative stereochemical assignments of the side-chain double bonds in **2** and **3**, were elucidated through comprehensive spectroscopic analysis and in comparison with the literature’s data. All isolated compounds were evaluated for their anti-leukaemic activities against human leukaemia cell lines K562 and RS4;11 using the MTT assay. Compounds **4**, **6**, **7**, and **9**–**12** exhibited notable cytotoxic activities against both RS4;11 and K562 cell lines, with IC_50_ values ranging from 5.02 ± 2.33 to 31.85 ± 0.50 μM. The results of Western blotting and Annexin V-FITC/PI staining elucidated that compounds **4**, **7**, and **10** could induce apoptosis in both RS4;11 cells and K562 cells.

## 1. Introduction

Cancer is one of the leading causes of death worldwide and poses a major challenge to public health systems [1]. Among haematological diseases, leukaemia represents a malignant proliferation of haematopoietic stem or progenitor cells in bone marrow, resulting in the accumulation of undifferentiated leukocytes and subsequent immune and hematologic dysfunctions [2]. According to the French–American–British classification systems, leukaemia can be categorised as acute myeloid leukaemia (AML), chronic myeloid leukaemia (CML), acute lymphoblastic leukaemia (ALL), and chronic lymphocytic leukaemia (CLL) [3]. Epidemiological data predict that the incidence of leukaemia will exceed 60,000 new cases in 2025 according to the National Cancer Institute (https://seer.cancer.gov/statfacts/html/leuks.html) (accessed on 15 April 2026) [4]. Although chemotherapy has been used to treat leukaemia for years, the severe adverse effects has limited its clinical application, such as hair loss, vomiting, hepatotoxicity, and neurological disorders, which are common causes of mortality in leukaemia patients [5,6]. Therefore, there is an urgent need to explore novel anti-leukaemia agents to improve clinical efficacy and enhance the quality of life for patients.

Covering nearly 70% of the Earth’s surface, marine ecosystems exhibit substantially greater biodiversity than terrestrial ecosystems [7]. The marine environment is characterised by extreme conditions such as high salinity, low oxygen, low temperature, and high pressure, which impose unique ecological pressures on marine organisms [8,9]. In response to these stresses, marine fungi have evolved distinct metabolic pathways, enabling them to produce structurally diverse and biologically potent secondary metabolites, many of which exhibit promising pharmacological activities [10,11].

*Aspergillus*, a representative genus of marine filamentous fungi, serves as a prolific producer of diverse secondary metabolites, including polyketides, alkaloids, terpenoids, peptides, and steroids [12]. Among them, alkaloids constitute the major class of secondary metabolites and can be subdivided into indole-diketopiperazine, indole-quinazoline, diketopiperazine, clavine-type ergot, spiro-heterocyclic γ-lactam, indole and indoline, amide, thiodiketopiperazine, and other alkaloid types [13]. Within this chemically diverse alkaloid pool, indole-diketopiperazine and spiro-heterocyclic γ-lactam frameworks represent two privileged structural families that recurrently emerge from *Aspergillus* species, particularly under marine or stress-induced fermentation conditions. Plinabulin exemplifies successful marine natural product drug development as a diketopiperazine-based microtubule-targeting agent. This compound was rationally designed using the structural framework of phenylahistin, a bioactive natural product isolated from the marine fungus *Aspergillus fumigatus*, through systematic structure–activity relationship studies to optimise its therapeutic potential [14]. Phase III clinical trial of plinabulin for non-small cell lung cancer has been completed [15]. Representative spiroγ-lactams from *A. fumigatus* have shown promising drug-like activities, including inhibition of the PCSK9–LDLR interaction and suppression of breast tumour recurrence in vivo, supporting their relevance as privileged scaffolds for drug discovery [13]. Although a huge amount of natural products have been isolated from *Aspergillus*, many secondary metabolite biosynthetic gene clusters remain silent and unexplored, indicating that this genus still represents a rich source of structurally diverse bioactive metabolites [16].

The overarching goal of this work was to uncover the chemical diversity and anti-leukaemia potential of secondary metabolites from the marine-derived fungus *Aspergillus fumigatus* SYPHU504. To this end, we set out to: (i) isolate and structurally characterise the major secondary metabolites produced by *A. fumigatus* SYPHU504; (ii) evaluate their in vitro cytotoxic activities against human chronic myeloid leukaemia (CML) K562 and acute lymphoblastic leukaemia (ALL) RS4;11 cell lines; and (iii) explore the apoptosis-inducing effects and apoptotic induction effect of representative active compounds. This study led to the isolation of a series of alkaloids with diverse scaffolds from *A. fumigatus* SYPHU504, several of which displayed notable cytotoxic activity against both K562 and RS4;11 cells, and were further investigated for their apoptosis-inducing properties.

## 2. Results

### 2.1. Structural Determination

**Figure 1:**
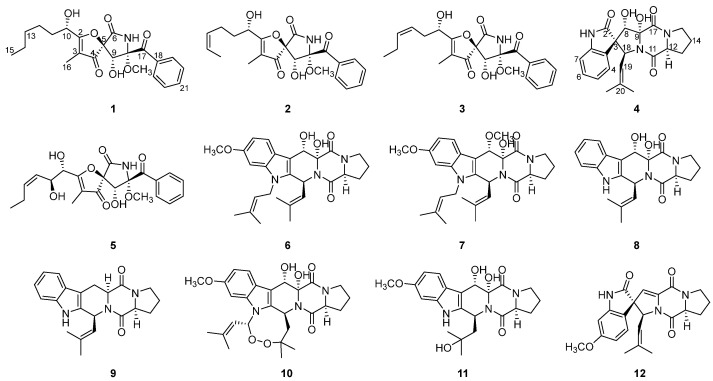
Chemical structures of the isolated compounds **1**–**12**.

Compound **1** (Figure 1) was obtained as a yellow amorphous powder (CH_3_OH). Its molecular formula was determined to be C_22_H_27_NO_7_ based on the (+)-HRESIMS ions at *m/z* 440.1691 [M + Na]^+^ (calcd. 440.1685 for C_22_H_27_NO_7_Na), indicating 10 degrees of unsaturation. Analysis of the ^1^H and ^13^C NMR data (Table 1) revealed the occurrence of the signals for a monosubstituted benzene ring at *δ*_H_ 8.35 (dd, *J* = 7.5, 1.5 Hz, 2H, H-19, 23), 7.64 (t, *J* = 7.5 Hz, 1H, H-21) and 7.50 (t, *J* = 7.5 Hz, 2H, H-20, 22); two oxymethine protons at *δ*_H_ 4.66 (m, 1H, H-10) and 4.50 (s, 1H, H-9); three sets of methylene protons at *δ*_H_ 1.81 (m, 2H, H-11), 1.47 (m, 2H, H-12) and 1.34 (m, 4H, H-13, 14); and one methoxy singlet at *δ*_H_ 3.34 (s, 3H, 8-OCH_3_). Furthermore, two methyl signals were detected at *δ*_H_ 1.75 (s, 3H, H-16, olefinic methyl) and 0.91 (t, *J* = 7.5 Hz, 3H, H-15, terminal methyl).

The ^13^C-NMR exhibited 22 carbon resonances, comparing three carbonyls (*δ*_C_ 199.4, 197.2 and 168.8); six aromatic carbons (*δ*_C_ 131.5, 134.9, 131.7 × 2 and 129.5 × 2); two olefinic carbons (*δ*_C_ 190.6 and 112.1); one methoxy carbon (*δ*_C_ 52.4); four oxygenated carbons (*δ*_C_ 93.8, 93.4, 76.3 and 68.9); four methylenes (*δ*_C_ 35.5, 32.8, 25.8 and 23.6); and two methyls (*δ*_C_ 14.3 and 5.5), supported by HSQC spectrum. These aforementioned spectroscopic features of **1** closely resembled those of pseurotin A [17], consistent with the presence of a spiro-heterocyclic *γ*-lactam core structure. The primary structural difference was the absence of the C-12/C-13 double bond found in pseurotin A, which is reduced in compound **1**.

**Figure 2:**
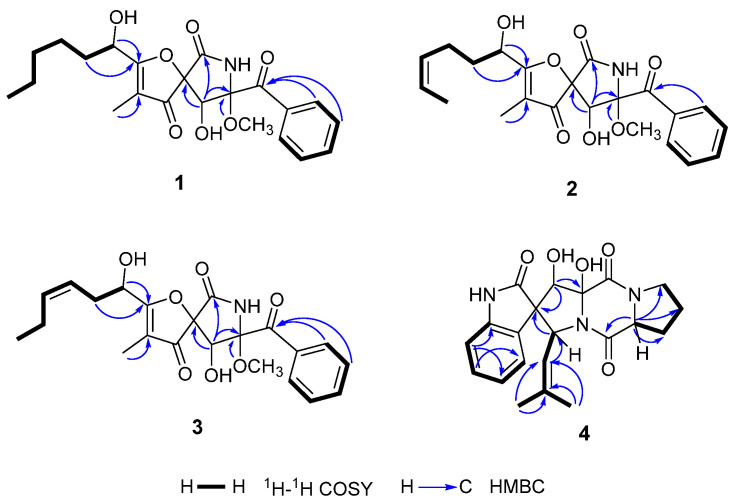
The ^1^H-^1^H COSY correlations and key HMBC correlations of compounds **1**–**4**.

The 2D constitutional structure of **1** was established through detailed analysis of its 2D NMR data (Figure 2). The ^1^H-^1^H COSY correlations spectrum revealed the presence of two key spin systems, including correlations of H-19/H-20/H-21/H-22/H-23, corresponding to a monosubstituted benzene ring; and the other comprising H-10/H-11/H-12/H-13/H-14/H-15, indicative of an aliphatic side chain. HMBC correlations from H-10/H-11 to C-2 suggested the side chain was connected to C-2. In addition, HMBC correlations from H-19, H-23, H-21 to C-17 confirmed the linkage of the monosubstituted phenyl group to C-17. Therefore, the 2D constitutional structure of **1** was established.

**Figure 3:**
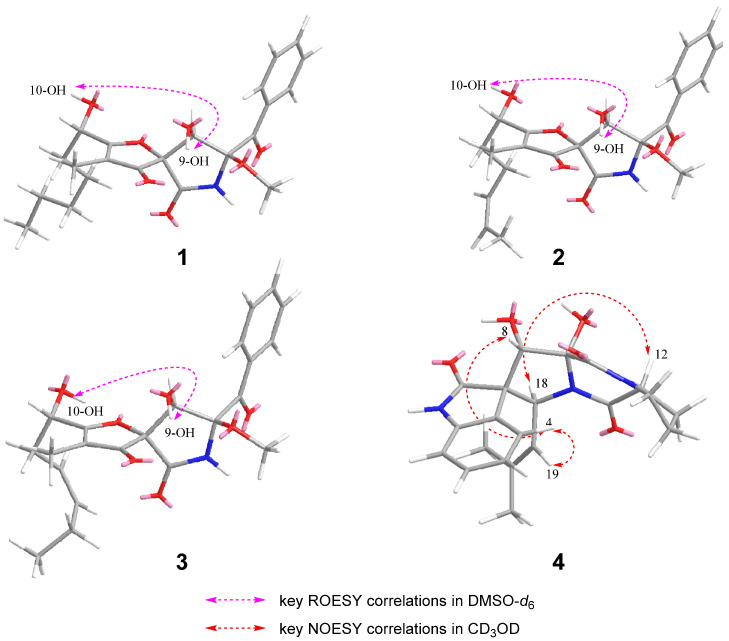
Key NOESY correlations of compounds **1**–**4**.

**Figure 4:**
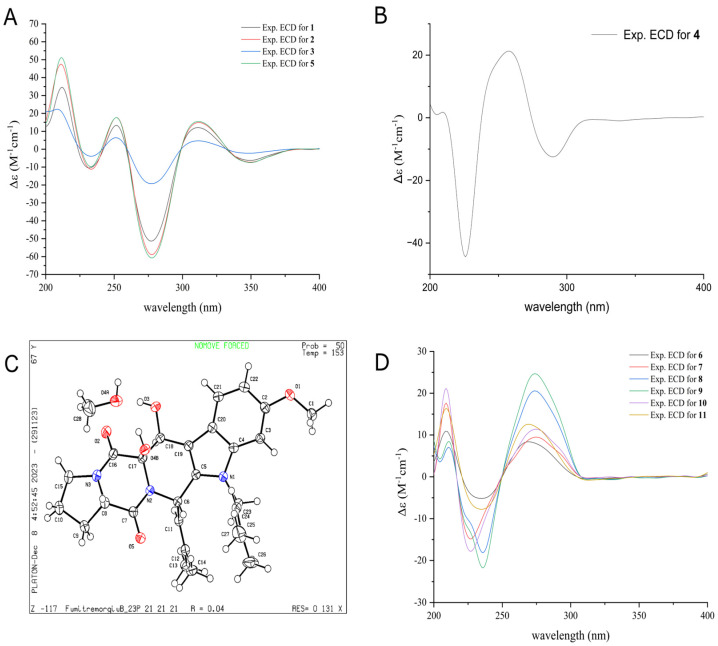
Experimental ECD spectra of compounds **1**–**11** (**A**,**B**,**D**); the X-ray crystal data for compound **6** (**C**).

The configuration of **1** was determined based on ROESY spectroscopy and comparison of its ECD spectrum with those of pseurotin A. Previous studies have shown that, in compounds bearing the same spiro-heterocyclic γ-lactam core, the coupling constant between H-9 and 9-OH in the ^1^H NMR spectrum serves as an indicator of the relative orientation between 9-OH and 8-OCH_3_. Specifically, a large coupling constant (*J* ∼ 10 Hz) is observed when 9-OH is oriented *cis* to 8-OCH_3_, whereas a small value (*J* ∼ 4 Hz) corresponds to a *trans* orientation [18]. Accordingly, the ^1^H NMR spectrum of compound 1 recorded in DMSO-d_6_ showed that 9*R*-OH [*δ*_H_ 6.35 (d, *J* = 9.0 Hz)] is cis-oriented to 8-OCH_3_. In the ROESY spectrum (measured in DMSO-*d*_6_), a cross-peak between 9-OH and 10-OH indicated that these protons possess the same spatial orientations (Figure 3). The **8*S*** configuration was assigned based on a negative Cotton effect at λ_max_ ∼ 280 nm, while the **5*S*** configuration was supported by a positive Cotton effect at λ_max_ ∼ 250 nm and a negative Cotton effect at λ_max_ ∼ 230 nm; these ECD trends were consistent with those reported in the literature by Takeshi Yamada et al. [19] (Figure 4). Thus, the absolute configurations of the chiral centres were supported as **5*S***, **8*S***, and **9*R***, and compound **1** was named as pseurotin N.

Compound **2** was obtained as a white amorphous powder (CH_3_OH). Its molecular formula was determined to be C_22_H_25_NO_7_ based on the (+)-HRESIMS ions at *m/z* 438.1532 [M + Na]^+^ (calcd. 438.1529 for C_22_H_25_NO_7_Na), indicating 11 degrees of unsaturation. The ^1^H and ^13^C NMR data (Table 1) of compound **2** closely resembled those of compound **1**, with the main difference being the presence of a double bond at C-13/C-14 [*δ*_H_ 5.47 (H-13, H-14); *δ*_C_ 135.1 (C-13), 126.9 (C-14)] in **2** instead of the two methylene groups [*δ*_H_ 1.34 (H-13, H-14); *δ*_C_ 32.8 (C-13), 23.6 (C-14)] observed in **1**. In the ROESY spectrum (measured in DMSO-*d*_6_), a cross-peak between 9-OH and 10-OH indicated that these protons possess the same spatial orientations (Figure 3). The **8*S*** configuration was assigned based on a negative Cotton effect at λ_max_ ∼ 280 nm, while the **5*S*** configuration was supported by a positive Cotton effect at λ_max_ ∼ 250 nm and a negative Cotton effect at λ_max_ ∼ 230 nm (Figure 4). The **9*R*** configuration was determined based on the coupling constant of 9-OH [*δ*_H_ 6.36 (d, *J* = 9.0 Hz)], which was the same as **1**. The geometry of the Δ^13,14^ double bonds could not be determined by coupling constant analysis, as H-13 and H-14 resonated as multiplets. Meanwhile, the *Z* configuration of the Δ^13,14^ double bond was supported by the ^13^C NMR chemical shift in the allylic carbon C-15 in **2** (*δ*_C_ 18.1), which is consistent with that reported for a related pseurotin derivative (*δ*_C_ 14.3) [20]. Furthermore, the negative-specific rotations observed for both compounds **2** and its derivatives (${\left[\alpha \right]}_{D}^{20}$-99.0 and -6.5, respectively) are consistent with the *Z* configuration assignment [20]. Thus, the absolute configurations of the chiral centres were tentatively assigned as **5*S***, **8*S***, **9*R***, **10*S***, and **13*Z***, and compound **2** was named pseurotin Y.

Compound **3** was obtained as a white amorphous powder (CH_3_OH). Its molecular formula was determined to be C_22_H_25_NO_7_ based on the (+)-HRESIMS ions at *m/z* 438.1533 [M + Na]^+^ (calcd. 438.1529 for C_22_H_25_NO_7_Na), indicating 11 degrees of unsaturation. The ^1^H and ^13^C NMR data (Table 1) of **3** closely resembled those of **2**, the main difference was the slight downfield or upfield shifts in some carbon and proton signals in the side chain, probably due to the double-bond group position being changed from Δ^13,14^ to Δ^12,13^. This was further confirmed by the ^1^H-^1^H COSY correlations of H-10/H-11/H-12/H-13/H-14/H-15 (Figure 2). Taking into consideration the multiplet peak pattern of H-12 and H-13, we tentatively determined the *Z* configuration of the Δ^12,13^ double bond by the ^13^C-NMR shift in C-14 in **3** (*δ*_C_ 21.6) compared to that of a related pseurotin derivative (*δ*_C_ 22.0) and the specific rotation in **2** (${\left[\alpha \right]}_{D}^{20}$-272.0) compared to that of a related pseurotin derivative (${\left[\alpha \right]}_{D}^{20}$-6.5) [18]. In the ROESY spectrum (measured in DMSO-*d_6_*), a cross-peak between 9-OH and 10-OH indicated that these protons possess the same spatial orientations (Figure 3). The **8*S*** configuration was assigned based on a negative Cotton effect at λ_max_ ∼ 280 nm, while the **5*S*** configuration was supported by a positive Cotton effect at λ_max_ ∼ 250 nm and a negative Cotton effect at λ_max_ ∼ 230 nm (Figure 4). The **9*R*** configuration was determined based on the coupling constant of 9-OH [*δ*_H_ 6.34 (d, *J* = 9.0 Hz)]. The absolute configuration of **3** could be tentatively determined as **5*S***, **8*S***, **9*R***, **10*S***, and **12*Z***. Thus, the completed structure of **3** was tentatively elucidated as depicted and named pseurotin S.

Compound **4** was obtained as a white amorphous powder (CH_3_OH). Its molecular formula was determined to be C_21_H_23_N_3_O_5_ based on the (+)-HRESIMS ions at *m/z* 420.1526 [M + Na]^+^ (calcd. 420.1535 for C_21_H_23_N_3_O_5_Na), indicating 12 degrees of unsaturation. Comparison of the NMR data (Table 2) of **4** with those of spirotryprostatin F from the marine-derived fungus *Aspergillus fumigatus* KMM 4631 suggested that compound **4** shares a similar skeleton to the known compound spirotryprostatin F [21]. The main difference between them was the absence of the methoxy group on the benzene ring. The ^1^H-^1^H COSY spectrum showed correlations of H-4/H-5/H-6/H-7 supporting the presence of a 1,2-disubstituted benzene ring in compound **4** (Figure 2). Thus, the 2D constitutional structure for **4** was established based on detailed spectroscopic analysis. The relative configurations of compound **4** were determined by the NOESY experiments, which showed NOE correlations from H-8 to H-4/H-19, from H-12 to H-18 (Figure 3). The absolute configurations of compound **4** were determined to be **3*S***, **8*S***, **9*R***, **12*S***, and **18*S*** by comparing with the CD spectra of the known compound spirotryprostatin F (Figure 4). Thus, the structure of compound **4**, designated to be spirotryprostatin S, was determined to be an undescribed diketopiperazine.

Meanwhile, the other eight known compounds were identified as pseurotin A (**5**) [17], fumitremorgin B (**6**) [22], prenylcyclotryprostatin B (**7**) [23], cyclotryprostatin C (**8**) [24], demethoxyfumitremorgin C (**9**) [25], verruculogen (**10**) [26], verruculogen TR-2 (**11**) [27], and 6-methoxyspirotryprostatin B (**12**) [28] by comparing their NMR data (Supplementary Materials) to previous reports. The absolute configurations of these compounds were further determined by electronic circular dichroism (ECD) analysis. Single-crystal X-ray diffraction data were obtained for compound **6** (deposited with the Cambridge Crystallographic Data Centre, deposition number: CCDC 2558621), unambiguously establishing its absolute configuration as **8*S***, **9*R***, **12*S***, and **18*S***. The ECD spectra of compounds **7**–**11** displayed Cotton effects consistent with those of compound **6**, indicating that these analogues share the same absolute configuration (**8*S***, **9*R***, **12*S***, **18*S***).

### 2.2. Bioactivity Assay

**Figure 5:**
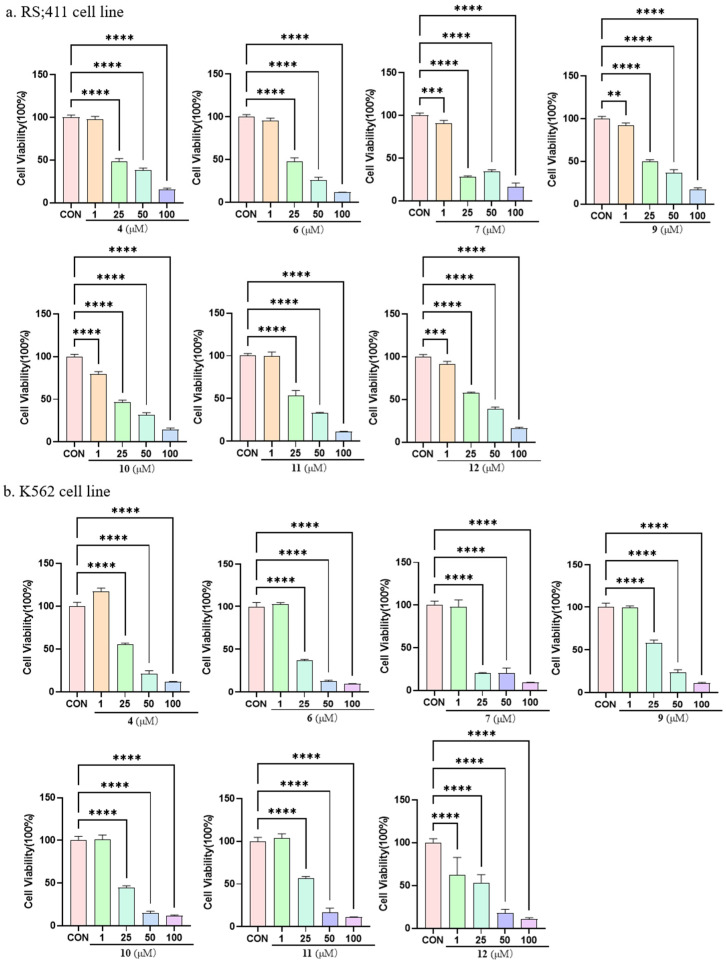
Anti-leukaemic effects of compounds **4**, **6**, **7**, and **9**–**12** on ALL cell line RS4;11 (**a**) and CML cell line K562 (**b**). ** *p* < 0.01, *** *p* < 0.001, **** *p* < 0.0001 vs. control.

An assay using 3-(4, 5-dimethylthiazol-2-yl)-2,5-diphenyltetrazolium bromide (MTT) was performed to evaluate the anti-leukaemic effects of compounds **1**–**12** in vitro by using the ALL cell line RS4;11 and the CML cell line K562. As shown in Figure 5, compounds **4**, **6**, **7**, and **9**–**12** exhibited significant dose-dependent anti-leukaemic activity against RS4;11 and K562 cell lines at the tested concentrations, with IC_50_ values ranging from 12.34 ± 0.92 to 31.85 ± 0.50 μM for RS4;11 and from 5.02 ± 2.33 to 28.87 ± 1.02 μM for K562 (Table 3).

### 2.3. Apoptotic Induction Effect of Compounds ***4***, ***7***, and ***10***

**Figure 6:**
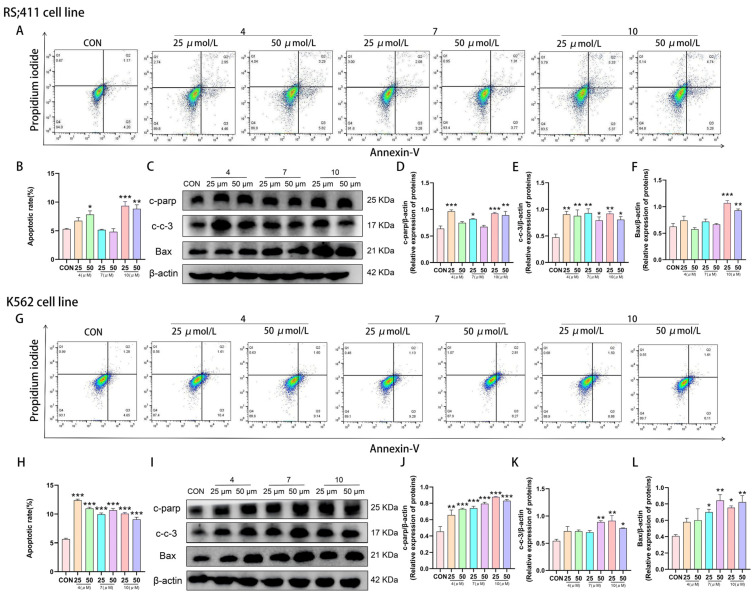
Compounds **4**, **7**, and **10** can induce apoptosis in RS4;11 cells and K562 cells. Graphs show (**A**,**G**) representative flow cytometry examination of Annexin V-PI double-staining; (**B**,**H**) the quantification of apoptosis rates; (**C**,**I**) the representative images of Western blotting; and the quantitative statistics of (**D**,**J**) cleaved PARP, (**E**,**K**) cleaved caspase-3, and (**F**,**L**) Bax. Data were shown as mean ± S.E., n = 3 independent tests. Significant differences were compared by using an independent *t*-test. * *p* < 0.05; ** *p* < 0.01; *** *p* < 0.001 vs. control group.

Based on the IC_50_ values obtained from the MTT assays, compounds **4**, **7**, and **10** were selected for the apoptotic induction effect of their anti-leukaemic effects. Flow cytometric analysis using Annexin V-FITC/PI staining revealed that treatment with compounds **4**, **7**, and **10** significantly increased the percentages of apoptotic cells in both RS4;11 and K562 cell lines compared with the control group (Figure 6A,B,G,H). Consistent with these findings, Western blotting analysis showed that compounds **4**, **7**, and **10** markedly upregulated the expression levels of key apoptosis-related proteins, including the pro-apoptotic protein Bax, as well as the executioner proteins cleaved PARP and cleaved caspase-3, in RS4;11 and K562 cells (Figure 6C–F,I–L). Collectively, these results indicate that compounds **4**, **7**, and **10** exert their cytotoxic effects by inducing apoptosis in RS4;11 and K562 cells.

## 3. Materials and Methods

### 3.1. Standardised Experimental Procedures

Optical rotations were measured on a MCP 200 polarimeter (Anton Paar, Shanghai, China). The spectra were recorded on a Bruker Avance 600 MHz spectrometer operating at 600 MHz for ^1^H and 150 MHz for ^13^C (Billerica, MA, USA). HRESIMS were recorded on a Xevo G2-XS QTof MS spectrometer (Waters Corporation, Milford, MA, USA). Analytical UPLC were performed on a Essentia LC-16 liquid chromatography system (Shimadzu, Kyoto, Japan) with an Agilent Extend-C18 column (5 μm, 4.6 × 250 mm). Preparative HPLC purifications were performed on a LC-20AT liquid chromatography system (Shimadzu, Kyoto, Japan) with a column YMC-Pack ODS-A (5 μm, 250 × 10 mm). TLC was performed on GF254 plates (Qingdao Marine Chemical Factory, Qingdao, China). Column chromatography (CC) was performed using silica gel (200–300 mesh, Qingdao Marine Chemical Factory, Qingdao, China), Sephadex LH-20 (GE Healthcare, Chicago, IL, USA), or ODS (40–63 μm, Merck, Darmstadt, Germany). All reagents used in the separation process were of analytical grade (AR, Sinopharm Chemical Reagent Co., Ltd., Shanghai, China). All reagents used in HPLC were of chromatographic grade (HPLC, Concord Technology, Tianjin, China).

### 3.2. Fungal Material

The fungal strain, *Aspergillus fumigatus* SYPHU504 (deposited at the Guangdong Microbial Culture Collection Centre (GDMCC), Guangzhou, China, under the accession number 65055), was isolated from the surface of a marine sponge sample collected from the South China Sea (20°36′2.212″ N, 109°41′50.523″ E). It was identified by the morphology and by analysis of the ITS regions of its rDNA (GenBank accession no. PP600218). The voucher specimen was deposited in our laboratory at −80 °C.

### 3.3. Extraction and Isolation

The fungal strain was grown on potato dextrose agar at 27 °C for 6 days. Then the mycelial agar plugs were transferred to 500 mL Erlenmeyer flasks containing 200 mL of potato dextrose broth, which were then incubated on a rotary shaker at 180 rpm and 27 °C for 3 days. Then, the seed liquid was spread in 1 L Erlenmeyer flasks (30 flasks, 160 g rice and 200 mL water in each Erlenmeyer flask). The flasks were incubated at 28 °C for 30 days.

The fermented medium was extracted three times with EtOAc, and was then concentrated under reduced pressure to produce an EtOAc extract (95.0 g), which was loaded on silica gel column chromatography and eluted with petroleum ether/EtOAc in gradient eluent (100:0–0:100 *v*/*v*), obtaining eight fractions Fr.1-Fr.8. Fr.2 was purified by silica gel (CH_2_Cl_2_/MeOH, 100:0–0:100 *v*/*v*), ODS (MeOH-H_2_O, 2:8–9:1, *v*/*v*), and semipreparative HPLC (MeOH-H_2_O, 65%, isocratic model) to yield compounds **6** (130 mg), **7** (5 mg), and **10** (15 mg). Fr.3 was purified by silica gel (CH_2_Cl_2_/MeOH, 100:0–0:100 *v*/*v*), ODS (MeOH-H_2_O, 2:8–9:1, *v*/*v*), and semipreparative HPLC (MeOH-H_2_O, 69%, isocratic model) to yield compounds **4** (3 mg), **8** (15 mg), and **9** (15 mg). Fr.4 was purified by silica gel (CH_2_Cl_2_/MeOH, 100:0–0:100 *v*/*v*), ODS (MeOH-H_2_O, 2:8–9:1, *v*/*v*), and semipreparative HPLC (MeOH-H_2_O, 72%, isocratic model) to yield compound **1** (3 mg). Fr.5 was purified by silica gel (CH_2_Cl_2_/MeOH, 100:0–0:100 *v*/*v*), ODS (MeOH-H_2_O, 2:8–9:1, *v*/*v*), and semipreparative HPLC (MeOH-H_2_O, 38%, isocratic model) to yield compounds **2** (1 mg) and **3** (2 mg); by semipreparative HPLC (MeOH-H_2_O, 42%, isocratic model) to yield compound **5** (30 mg); and by semipreparative HPLC (MeOH-H_2_O, 60%, isocratic model) to yield compounds **11** (4 mg) and **12** (5 mg).

### 3.4. Spectroscopic Data of Compounds

Pseurotin N (**1**): a yellow amorphous powder; ${\left[\alpha \right]}_{D}^{20}$-50.3 (*c* 0.1, CH_3_OH); ^1^H and ^13^C NMR, Table 1; (+)-HRESIMS *m/z* 440.1691 [M + Na]^+^ (calcd. 440.1685 for C_22_H_27_NNaO_7_).

Pseurotin Y (**2**): a white amorphous powder; ${\left[\alpha \right]}_{D}^{20}$-99.0 (*c* 0.2, CH_3_OH); ^1^H and ^13^C NMR, Table 1; (+)-HRESIMS *m/z* 438.1532 [M + Na]^+^ (calcd. 438.1529 for C_22_H_25_NNaO_7_).

Pseurotin S (**3**): a white amorphous powder; ${\left[\alpha \right]}_{D}^{20}$-272.0 (*c* 0.2, CH_3_OH); ^1^H and ^13^C NMR, Table 1; (+)-HRESIMS *m/z* 438.1533 [M + Na]^+^ (calcd. 438.1529 for C_22_H_25_NNaO_7_).

Spirotryprostatin S (**4**): a white amorphous powder; ${\left[\alpha \right]}_{D}^{20}$-486.0 (*c* 0.2, CH_3_OH); ^1^H and ^13^C NMR, Table 2; (+)-HRESIMS *m/z* 420.1526 [M + Na]^+^ (calcd. 420.1535 for C_21_H_23_N_3_NaO_5_).

### 3.5. Cell Culture

RS4;11 and K562 cells were cultured in RPMI 1640 medium containing 10% (*v*/*v*) of heated inactivated foetal bovine serum, 100 U/mL penicillin, 100 μg/mL streptomycin, and 1 mmol/L glutamine. The cells were cultured in a sterile culture dish and inoculated in a cell incubator containing 5% CO_2_ at 37 °C.

### 3.6. MTT Assay

The isolated compounds (**1**–**12**) were evaluated for their antiproliferative activity on human leukaemia cell lines RS4;11 and K562 in vitro by using the MTT assay. In brief, RS4;11 and K562 cells were plated in 96-well plates at a density of 3 × 10^4^ cells/mL and incubated at 37 °C for 48 h in the incubator with CO_2_. Then, 10 µL of serum-free medium containing various concentrations (1, 25, 50, and 100 μmol/L) of compounds were added to the wells and incubated at 37 °C for another 72 h. After adding 100 µL of MTT and incubated at 37 °C for another 3–4 h, the 96-well plates were centrifuged (2000 *g*, 15 min) and the supernatant were removed. Then we added 100 µL of DMSO to each well and shook these gently. After 10 min, the absorbance at 490 was measured by using an enzyme metre. The vehicle we used was DMSO and the final solvent concentration of DMSO was 0.001 μmol/L. Each treatment concentration of each compound has 6 technical replicate wells. The MTT assay of these compounds was also biologically replicated three times. The limitation of this experiment is that we did not use a positive control. The IC_50_ values of these compounds were calculated using the software Graphpad 9.5.

### 3.7. Flow Cytometric Apoptosis Assay

RS4;11 and K562 cells were divided into a control group (CON) and compound groups of **4**, **7**, and **10** (25 μmol/L and 50 μmol/L). After treatment for 72h, cells were collected and washed using PBS. Then the cells were double-stained with Annexin V-FITC and propidium iodide (PI) in the dark. Fluorescence signals were examined by using flow cytometry. The apoptotic percentage were calculated as the sum of early and late apoptotic cells.

### 3.8. Western Blotting Analysis

Total cellular proteins were extracted by using RIPA lysis buffer. After quantification, proteins were separated using SDS-PAGE and electroblotted onto PVDF membranes. Then, the membranes were blocked with 5% non-fat milk. The membranes were then incubated with primary antibodies against cleaved parp (c-parp, 25 kDa), activated cleaved Caspase-3 (c-c-3, 17 kDa), and the pro-apoptotic protein Bax (21 kDa), with β-actin (42 kDa) as the loading control. After incubation with secondary antibodies, protein bands were visualised using the ECL chemiluminescence. Band intensities were quantified using the ImageJ software (1.50b). Experiments were repeated three times for statistical significance analysis.

### 3.9. Data Analysis

Data were analysed using GraphPad Prism and expressed as means ± standard error of means. Differences between control and exposed groups were analysed using one-way ANOVA combined with Tukey’s test, with *p* < 0.05 considered statistically significant.

## 4. Conclusions

In this study, three undescribed γ-lactam alkaloids (**1**–**3**) and one novel diketopiperazine (**4**), along with eight other known compounds (**5**–**12**), were isolated and structurally elucidated from an ethyl acetate extract of the marine-derived fungus *Aspergillus fumigatus* SYPHU504. It is worth noting that the unambiguous determination of the side-chain double-bond geometries in compounds **2** and **3** presented a challenge. In their ^1^H NMR spectra, the olefinic protons appeared as complex multiplets due to signal overlapping, which precluded the direct extraction of characteristic coupling constants (*J* values) for configuration assignment. To address this limitation, we turned to the ^13^C NMR data, specifically focusing on the chemical shifts in the allylic carbons, which are highly sensitive to the steric environment of adjacent double bonds. By systematically comparing these carbon chemical shifts with those of structurally related analogues reported in the literature, the geometries of the double bonds were tentatively deduced. Furthermore, the overall stereochemical assignments were corroborated by the consistency of the negative optical rotation values with known reference compounds. Although the assignment relies on empirical chemical shift trends and the literature’s comparisons rather than direct proton–proton coupling analysis, the convergent evidence strongly supports the proposed configurations, which are thus tentatively assigned as E/Z.

The anti-leukaemic potential of these compounds was evaluated in vitro using the MTT assay, revealing that compounds **4**, **6**, **7**, and **9**–**12** exhibited cytotoxic activity against both RS4;11 (IC_50_ = 12.34 ± 0.92 to 31.85 ± 0.50 μM) and K562 (IC_50_ = 5.02 ± 2.33 to 28.87 ± 1.02 μM) leukaemia cell lines. Furthermore, representative compounds (**4**, **7**, and **10**) were selected for mechanistic studies; flow cytometry and Western blotting analyses demonstrated that these compounds significantly increased apoptotic cell populations and upregulated key pro-apoptotic and executioner proteins (Bax, cleaved PARP, and cleaved caspase-3) in RS4;11 cells, indicating that they can induce apoptosis in these two cell lines.

Notably, the γ-lactam alkaloids showed no significant cytotoxic activity under the tested conditions, whereas the diketopiperazine alkaloids exhibited more potent cytotoxicity with a clear dose-dependent response within the tested concentration range.

In conclusion, while the in vitro results—including IC_50_ values, apoptosis induction, and regulation of pro-apoptotic proteins—demonstrate the potential of these diketopiperazine alkaloids, the lack of positive control of the MTT assay and the in vivo efficacy and safety data remain a major limitation of this study. Consequently, these findings should be interpreted as a preliminary biological evaluation, and further animal model studies are required before any definitive statements can be made regarding their broader therapeutic potential.

## Figures and Tables

**Table 1:** The ^1^H NMR (600 MHz) and ^13^C NMR (150 MHz) data for compounds **1**–**3** in CD_3_OD.

NO.	1		2		3	
	*δ*_C_, Type	*δ*_H_ (J in Hz)	*δ*_C_, Type	*δ*_H_ (J in Hz)	*δ*_C_, Type	*δ*_H_ (J in Hz)
2	190.6, C		190.4, C		190.1, C	
3	112.1, C		112.3, C		112.3, C	
4	199.4, C		199.4, C		199.4, C	
5	93.8, C		93.8, C		93.8, C	
6	168.8, C		168.8, C		168.8, C	
8	93.4, C		93.4, C		93.4, C	
9	76.3, CH	4.50, s	76.3, CH	4.50, s	76.4, CH	4.51, s
10	68.9, CH	4.66, m	68.2, CH	4.65, t (6.8)	69.0, CH	4.65, t (6.8)
11	35.5, CH_2_	1.81, m	29.1, CH_2_	1.85, m	33.4, CH_2_	2.58, m
12	25.8, CH_2_	1.47, m	35.3, CH_2_	2.15, m	124.1, CH	5.47, m
13	32.8, CH_2_	1.34, m	135.1, CH	5.47, m	136.0, CH	5.47, m
14	23.6, CH_2_	1.34, m	126.9, CH	5.47, m	21.6, CH_2_	2.09, m
15	14.3, CH_3_	0.91, t (7.5)	18.1, CH_3_	1.63, m	14.5, CH_3_	0.96, s
16	5.5, CH_3_	1.75, s	5.5, CH_3_	1.74, s	5.6, CH_3_	1.74, s
17	197.2, C		197.2, C		197.2, C	
18	134.9, C		131.7, C		134.9, C	
19	131.7, CH	8.35, dd (7.5, 1.5)	131.3, CH	8.35, dd (7.9, 1.0)	131.7, CH	8.35, dd (7.9, 1.0)
20	129.5, CH	7.50, t (7.5)	129.5, CH	7.50, t (7.9)	129.5, CH	7.50, t (7.9)
21	135.1, CH	7.64, t (7.5)	134.9, CH	7.64, t (7.9)	135.1, CH	7.64, t (7.9)
22	129.5, CH	7.50, t (7.5)	129.5, CH	7.50, t (7.9)	129.5, CH	7.50, t (7.9)
23	131.7, CH	8.35, dd (7.5, 1.5)	131.3, CH	8.35, dd (7.9, 1.0)	131.7, CH	8.35, dd (7.9, 1.0)
8-OCH_3_	52.4, CH_3_	3.34, s	52.4, CH_3_	3.34, s	52.4, CH_3_	3.34, s
9-OH ^1^		6.35, d (9.0)		6.36, d (9.0)		6.34, d (9.0)
10-OH ^1^		5.62, d (5.5)		5.67, d (5.4)		5.74, d (5.5)

^1^ Measured in DMSO-*d*_6_.

**Table 2:** The ^1^H NMR (600 MHz) and ^13^C NMR (150 MHz) data for compound **4** in CD_3_OD.

NO.	*δ*_C_, Type	*δ*_H_ (J in Hz)	NO.	Δ_c_, Type	*δ*_H_ (J in Hz)
2	182.9, C		12	62.0, CH	4.66 (m)
3	63.6, C		13	28.9, CH_2_	2.37 (m)
3a	130.0, C		14	24.2, CH_2_	2.04 (m)
4	128.2, CH	7.20 (dd, 7.6, 1.2)	15	46.2, CH_2_	3.55 (m)
5	123.4, CH	7.04 (td, 7.6, 1.2)	17	166.8, C	
6	127.9, CH	7.26 (td, 7.6, 1.2)	18	58.6, CH	4.88 (m)
7	110.9, CH	6.90 (dd, 7.6, 1.2)	19	123.2, CH	4.88 (m)
7a	144.2, C		20	139.5, C	
8	75.9, CH	5.01 (s)	21	25.3, CH_3_	1.60 (s)
9	88.7, C		22	18.0, CH_3_	1.06 (s)
11	171.5, C				

**Table 3:** Anti-leukaemic effects of compounds **4**, **6**, **7**, and **9**–**12**.

Compound	IC_50_ (μM)	
	RS4;11	K562
**4**	26.10 ± 1.27	27.52 ± 0.63
**6**	22.66 ± 1.73	17.95 ± 0.34
**7**	12.34 ± 0.92	10.31 ± 1.18
**9**	24.29 ± 0.92	28.87 ± 1.02
**10**	13.28 ± 1.04	21.42 ± 0.99
**11**	28.40 ± 1.87	27.34 ± 0.57
**12**	31.85 ± 0.50	5.02 ± 2.33

## Data Availability

All the data are included within the article.

## Supplementary Materials

The following supporting information can be downloaded at: https://www.mdpi.com/article/10.3390/md24070247/s1, Figure S1. HRESIMS spectrum of 1. Figures S2–S4. 1D-NMR spectrum of **1** (CD_3_OD, 600 MHz for ^1^H, 150 MHz for ^13^C). Figures S5–S8. 2D-NMR spectrum of **1** (CD_3_OD). Figure S9. HRESIMS spectrum of **2**. Figures S10–S12. 1D-NMR spectrum of **2** (DMSO-d_6_, 600 MHz for ^1^H, 150 MHz for ^13^C). Figures S13–S15. 2D-NMR spectrum of **2** (DMSO-d_6_). Figure S16. HRESIMS spectrum of **3**. Figures S17–S19. 1D-NMR spectrum of **3** (CD_3_OD, 600 MHz for ^1^H, 150 MHz for ^13^C). Figures S20–S23. 2D-NMR spectrum of **3** (DMSO-d_6_). Figure S24. HRESIMS spectrum of **4**. Figures S25–S26. 1D-NMR spectrum of **3** (DMSO-d_6_, 600 MHz for ^1^H, 150 MHz for ^13^C). Figures S27 and S28. 2D-NMR spectrum of **4** (CD_3_OD). Figures S29 and S30. 1D-NMR spectrum of **5** (CD_3_OD, 600 MHz for ^1^H, 150 MHz for ^13^C). Figures S31 and S32. 1D-NMR spectrum of **6** (CDCl_3_, 600 MHz for ^1^H, 150 MHz for ^13^C). Figure S33. X-ray diffraction data of 6. Figures S34 and S35. 1D-NMR spectrum of **7** (CD_3_OD, 600 MHz for ^1^H, 150 MHz for ^13^C). Figures S36 and S37. 1D-NMR spectrum of **8** (CDCl_3_, 600 MHz for ^1^H, 150 MHz for ^13^C). Figures S38 and S39. 1D-NMR spectrum of **9** (CDCl_3_, 600 MHz for ^1^H, 150 MHz for ^13^C). Figures S40 and S41. 1D-NMR spectrum of **10** (CDCl_3_, 600 MHz for ^1^H, 150 MHz for ^13^C). Figures S42 and S43. 1D-NMR spectrum of **11** (CD_3_OD, 600 MHz for ^1^H, 150 MHz for ^13^C). Figures S44 and S45. 1D-NMR spectrum of **12** (CD_3_OD, 600 MHz for ^1^H, 150 MHz for ^13^C).
