# Supplementary material for: Isolation and Characterisation of Alkaloids from Marine-Derived Aspergillus fumigatus SYPHU504 with Antiproliferative Activity
Source: Mar Drugs. 2026 Jul 16;24(7):247. doi: 10.3390/md24070247 (PMC13413414; doi:10.3390/md24070247)
Supplement: Supplementary file 1 [file marinedrugs-24-00247-s001.zip › marinedrugs-4347235-supplementary.pdf]

Supporting Information

Isolation and Characterisation of Alkaloids from Marine-Derived *Aspergillus fumigatus* SYPHU504 with Antiproliferative Activity

Xuelei Zhang [1,†], Yingshu Yu [1,†], Kai Liu [2], Yonghong Liu [1,*], Hong Zhang [3,*] and Jiao Xiao [1,4,*]

[1] Wuya College of Innovation, Shenyang Pharmaceutical University, Shenyang 110016, China; 15248089084@163.com (X.Z.); yuyingshu98@163.com (Y.Y.)
[2] Guangxi Key Laboratory of Marine Drugs and Institute of Marine Drugs, Guangxi University of Chinese Medicine, Nanning 530200, China; kailiu@gxtcmu.edu.cn
[3] School of Clinical Pharmacy, Shenyang Pharmaceutical University, Shenyang 110016, China
[4] School of Traditional Chinese Materia Medica, Shenyang Pharmaceutical University, Shenyang 110016, China
* Correspondence: yonghongliu@scsio.ac.cn (Y.L.); song0688@sina.com (H.Z.); xj110121@126.com (J.X.)
† These authors contribute equally to this work.

Contents

Figure S1. HRESIMS spectrum of **1**. 3
Figure S2. ^{1}H-NMR spectrum of **1** (CD_3OD, 600 MHz). 3
Figure S3. ^{1}H-NMR spectrum of **1** (DMSO-d_6, 600 MHz). 4
Figure S4. ^{13}C-NMR spectrum of **1** (CD_3OD, 150 MHz). 4
Figure S5. ^{1}H-^{1}H COSY spectrum of **1** (CD_3OD). 5
Figure S6. HSQC spectrum of **1** (CD_3OD). 5
Figure S7. HMBC spectrum of **1** (CD_3OD). 6
Figure S8. ROESY spectrum of **1** (DMSO-d_6). 6
Figure S9. HRESIMS spectrum of **2**. 7
Figure S10. ^{1}H-NMR spectrum of **2** (CD_3OD, 600 MHz). 7
Figure S11. ^{1}H-NMR spectrum of **2** (DMSO-d_6, 600 MHz). 8
Figure S12. ^{13}C-NMR spectrum of **2** (CD_3OD, 150 MHz). 8
Figure S13. ^{1}H-^{1}H COSY spectrum of **2** (CD_3OD). 9
Figure S14. HMBC spectrum of **2** (CD_3OD). 9
Figure S15. ROESY spectrum of **2** (DMSO-d_6). 10
Figure S16. HRESIMS spectrum of **3**. 10
Figure S17. ^{1}H-NMR spectrum of **3** (CD_3OD, 600 MHz). 11
Figure S18. ^{1}H-NMR spectrum of **3** (DMSO-d_6, 600 MHz). 11
Figure S19. ^{13}C-NMR spectrum of **3** (CD_3OD, 150 MHz). 12
Figure S20. ^{1}H-^{1}H COSY spectrum of **3** (CD_3OD). 12
Figure S21. HSQC spectrum of **3** (CD_3OD). 13
Figure S22. HMBC spectrum of **3** (CD_3OD). 13
Figure S23. ROESY spectrum of **3** (DMSO-d_6). 14
Figure S24. HRESIMS spectrum of **4**. 14
Figure S25. ^{1}H-NMR spectrum of **4** (CD_3OD, 600 MHz). 15
Figure S26. ^{13}C-NMR spectrum of **4** (CD_3OD, 150 MHz). 15
Figure S27. ^{1}H-^{1}H COSY spectrum of **4** (CD_3OD). 16
Figure S28. NOESY spectrum of **4** (CD_3OD). 16
Figure S29. ^{1}H-NMR spectrum of **5** (CD_3OD, 600 MHz). 17
Figure S30. ^{13}C-NMR spectrum of **5** (CD_3OD, 150 MHz). 17
Figure S31. ^{1}H-NMR spectrum of **6** ($CDCl_3$, 600 MHz). 18
Figure S32. ^{13}C-NMR spectrum of **6** ($CDCl_3$, 150 MHz). 18
Figure S33. X-ray diffraction data of **6**. 20
Figure S34. ^{1}H-NMR spectrum of **7** (CD_3OD, 600 MHz). 20
Figure S35. ^{13}C-NMR spectrum of **7** (CD_3OD, 150 MHz). 21
Figure S36. ^{1}H-NMR spectrum of **8** ($CDCl_3$, 600 MHz). 21
Figure S37. ^{13}C-NMR spectrum of **8** ($CDCl_3$, 150 MHz). 22
Figure S38. ^{1}H-NMR spectrum of **9** ($CDCl_3$, 600 MHz). 22
Figure S39. ^{13}C-NMR spectrum of **9** ($CDCl_3$, 150 MHz). 23
Figure S40. ^{1}H-NMR spectrum of **10** ($CDCl_3$, 600 MHz). 23
Figure S41. ^{13}C-NMR spectrum of **10** ($CDCl_3$, 150 MHz). 24
Figure S42. ^{1}H-NMR spectrum of **11** (CD_3OD, 600 MHz). 24
Figure S43. ^{13}C-NMR spectrum of **11** (CD_3OD, 150 MHz). 25
Figure S44. ^{1}H-NMR spectrum of **12** (CD_3OD, 600 MHz). 25
Figure S45. ^{13}C-NMR spectrum of **12** (CD_3OD, 150 MHz). 26

Elemental Composition Report **Page 1**

Single Mass Analysis
Tolerance = 5.0 mDa / DBE: min = -1.5, max = 50.0
Element prediction: Off
Number of isotope peaks used for i-FIT = 3

Monoisotopic Mass, Even Electron Ions
774 formula(e) evaluated with 1 results within limits (up to 50 best isotopic matches for each mass)
Elements Used:
C: 22-22 H: 27-27 N: 0-200 O: 0-200 Na: 0-1
10
231011-2-121-2-yy-n12-70-1-4 10 (0.111) 1: TOF MS ES+ 3.06e+005

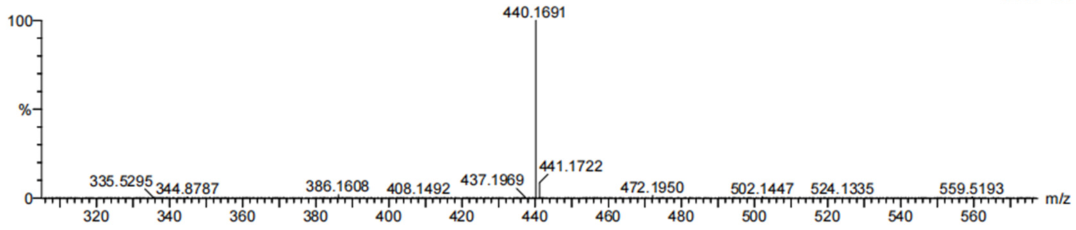

Minimum:				-1.5				
Maximum:		5.0	10.0	50.0				
Mass	Calc. Mass	mDa	PPM	DBE	i-FIT	Norm	Conf(%)	Formula
440.1691	440.1685	0.6	1.4	9.5	200.0	n/a	n/a	C22 H27 N O7 Na

Figure S1. HRESIMS spectrum of **1**.

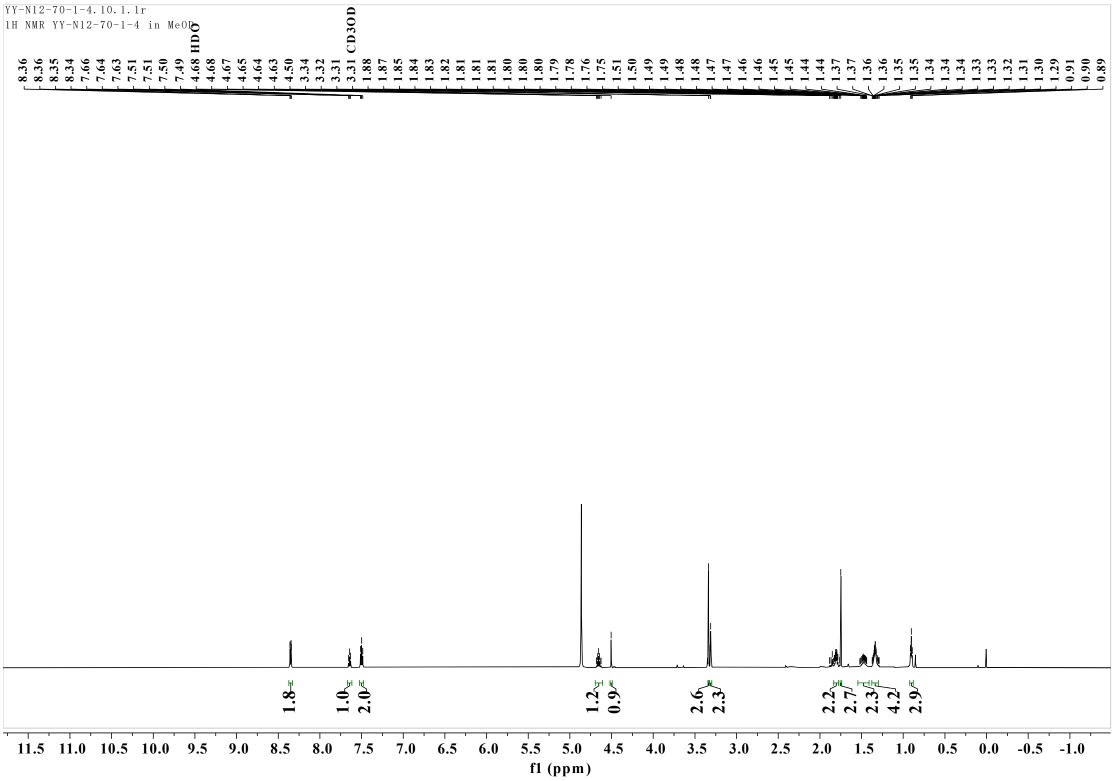

Figure S2. ^{1}H-NMR spectrum of **1** (CD_3OD, 600 MHz).

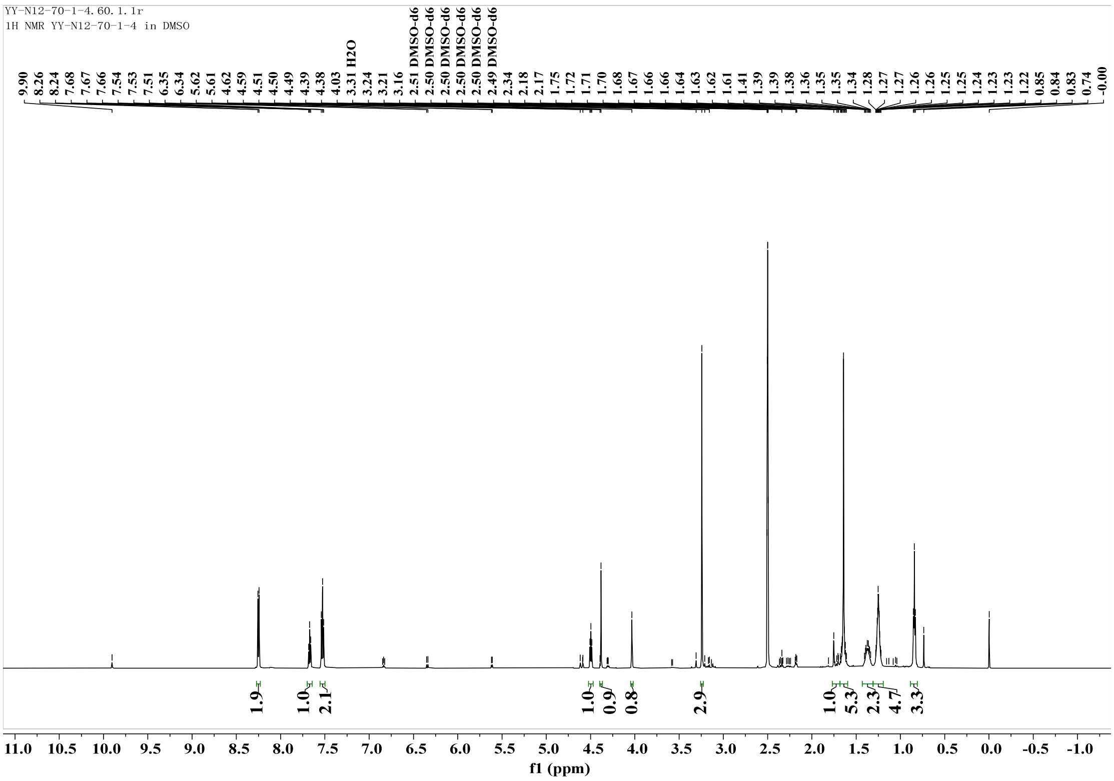

Figure S3. ^{1}H-NMR spectrum of **1** (DMSO-d_6, 600 MHz).

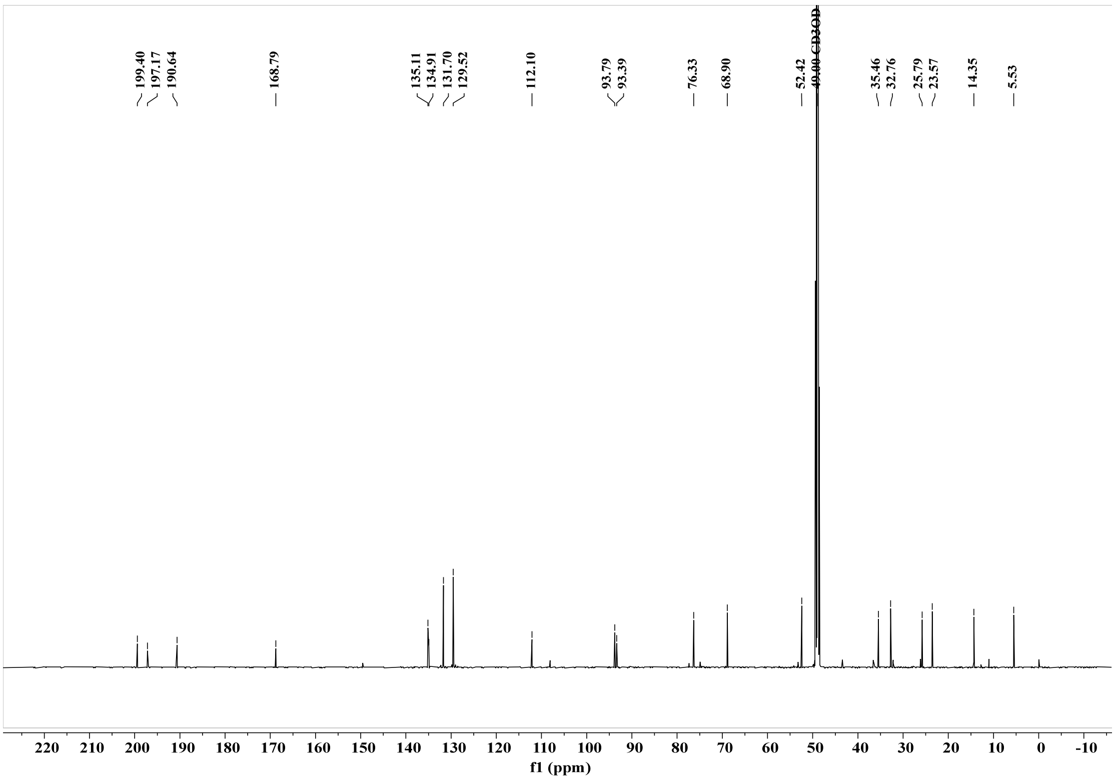

Figure S4. ^{13}C-NMR spectrum of **1** (CD_3OD, 150 MHz).

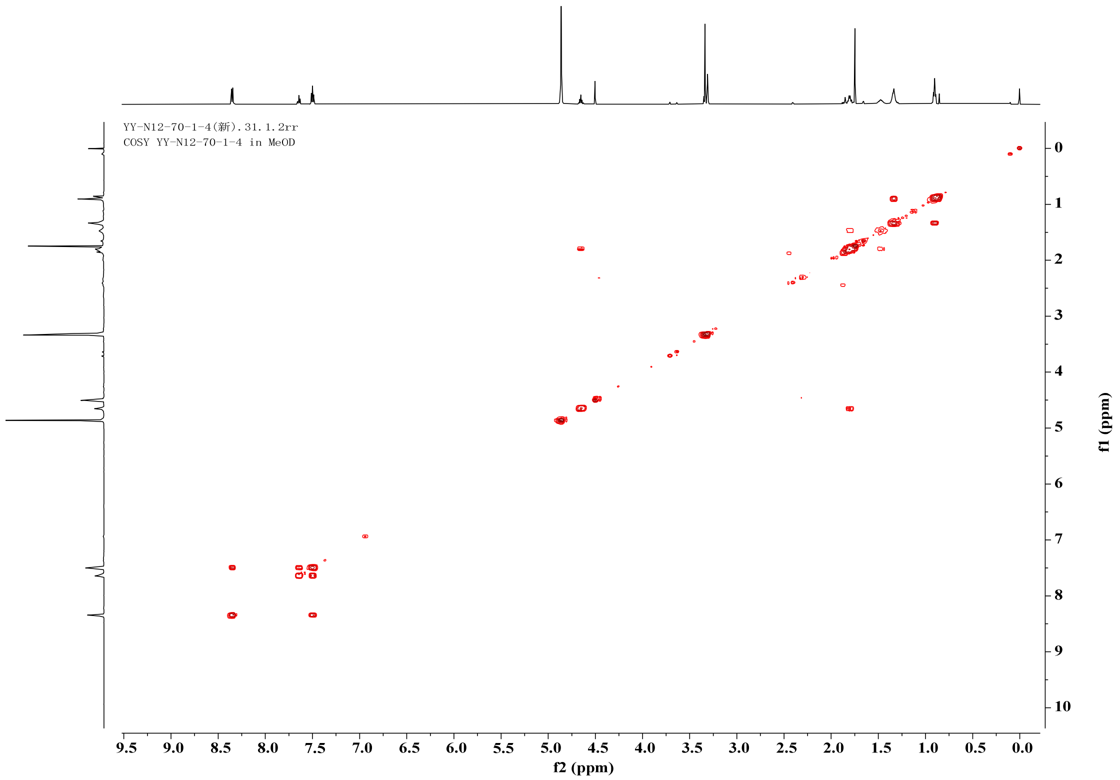

Figure S5. ^{1}H-^{1}H COSY spectrum of **1** (CD_3OD).

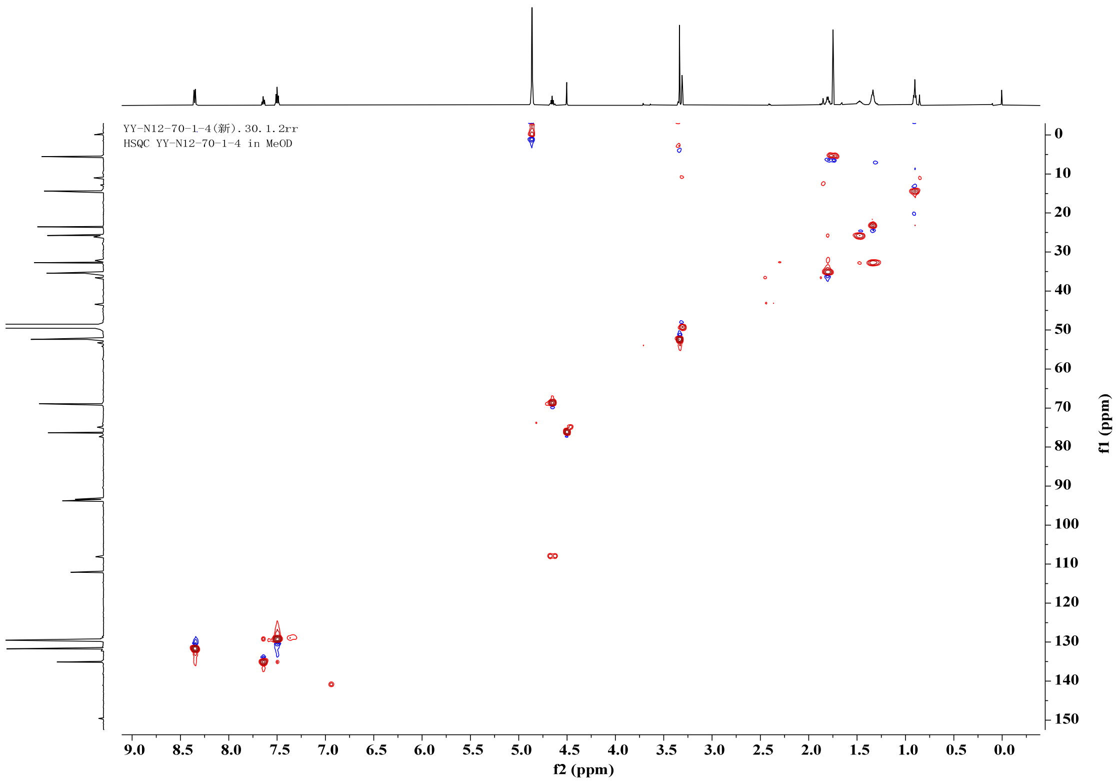

Figure S6. HSQC spectrum of **1** (CD_3OD).

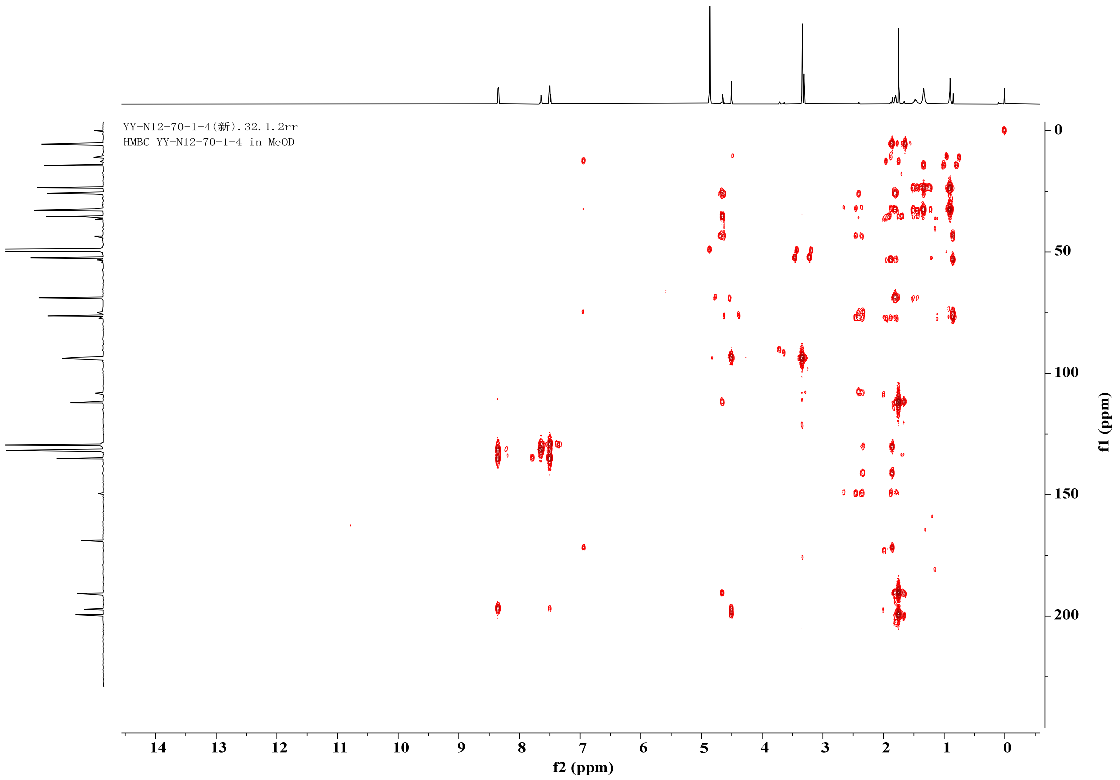

Figure S7. HMBC spectrum of **1** (CD_3OD).

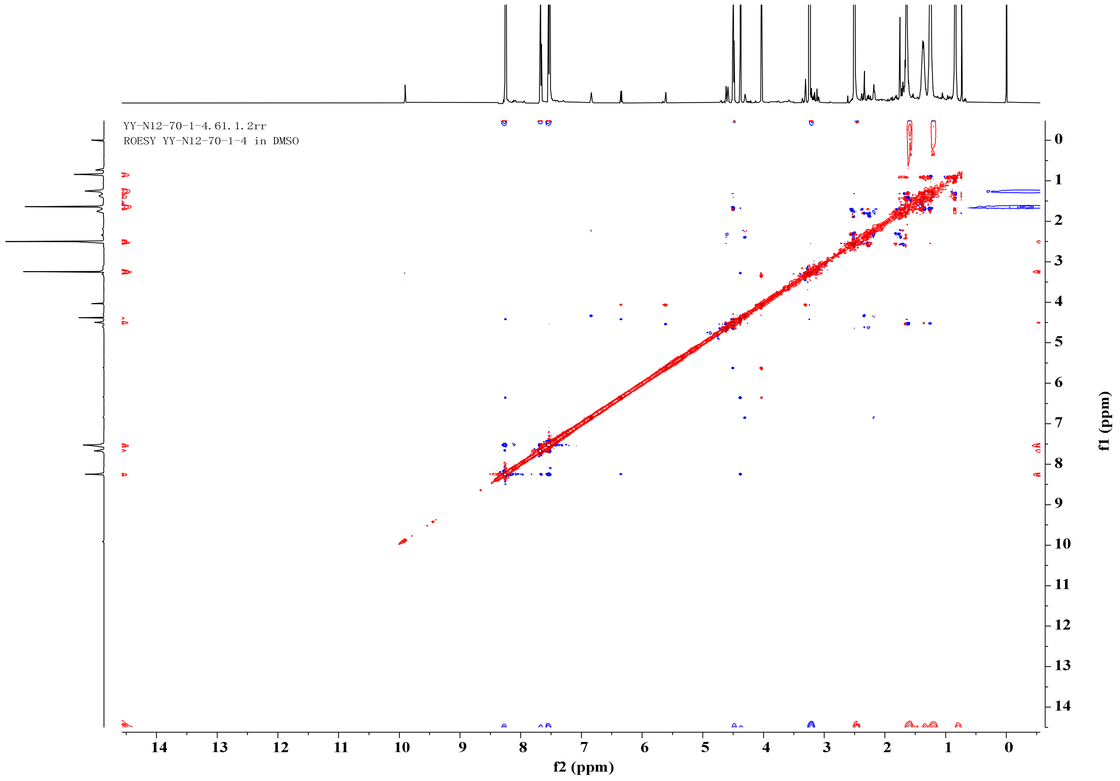

Figure S8. ROESY spectrum of **1** (DMSO-d_6).

Elemental Composition Report **Page 1**

Single Mass Analysis
Tolerance = 5.0 mDa / DBE: min = -1.5, max = 50.0
Element prediction: Off
Number of isotope peaks used for i-FIT = 3

Monoisotopic Mass, Even Electron Ions
403 formula(e) evaluated with 1 results within limits (up to 50 best isotopic matches for each mass)
Elements Used:
C: 22-22 H: 25-25 N: 0-10 O: 0-100 Na: 0-1
11
240327-6-196-5-zheng-4 9 (0.069) 1: TOF MS ES+ 2.58e+005

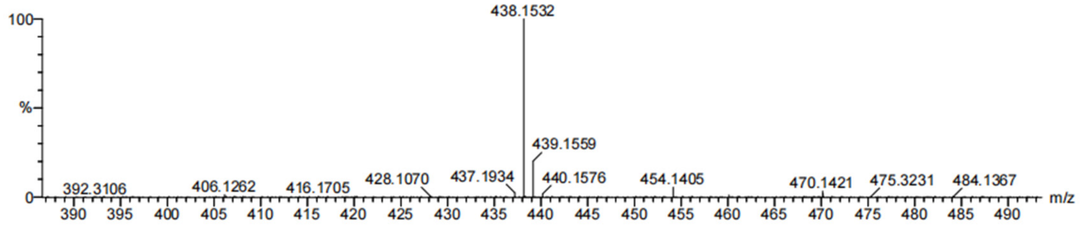

Minimum:				-1.5				
Maximum:		5.0	10.0	50.0				
Mass	Calc. Mass	mDa	PPM	DBE	i-FIT	Norm	Conf(%)	Formula
438.1532	438.1529	0.3	0.7	10.5	197.9	n/a	n/a	C22 H25 N O7 Na

Figure S9. HRESIMS spectrum of **2**.

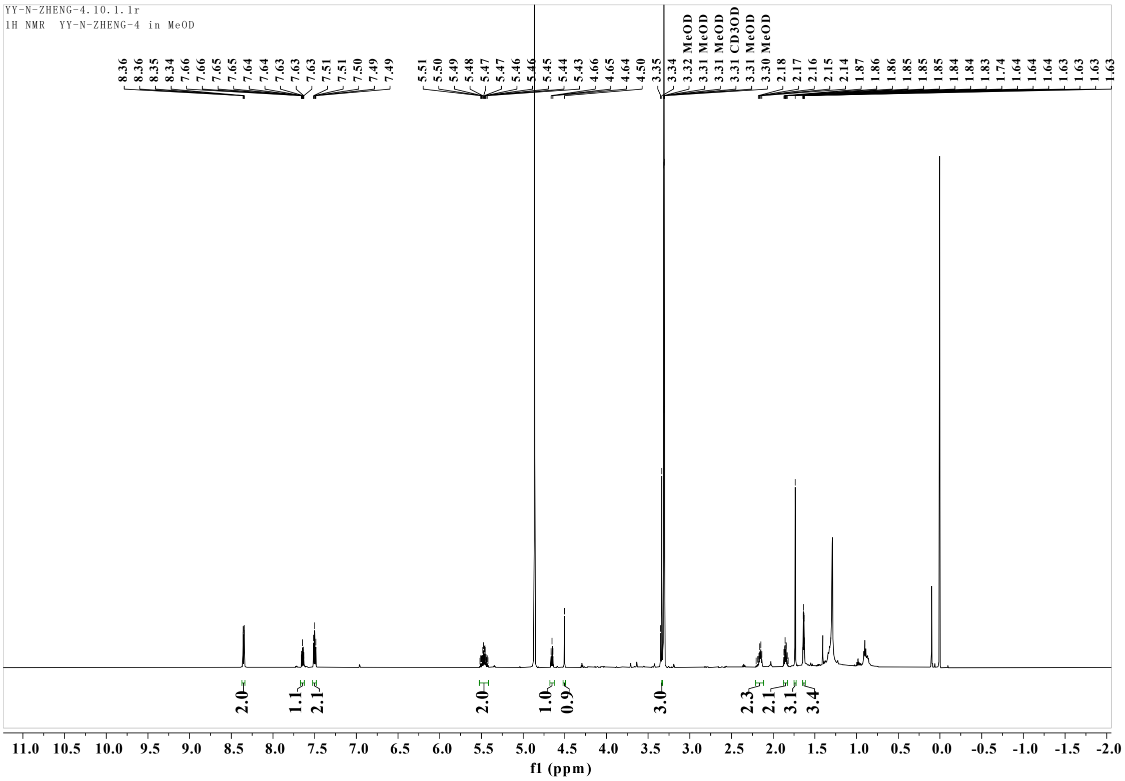

Figure S10. ^{1}H-NMR spectrum of **2** (CD_3OD, 600 MHz).

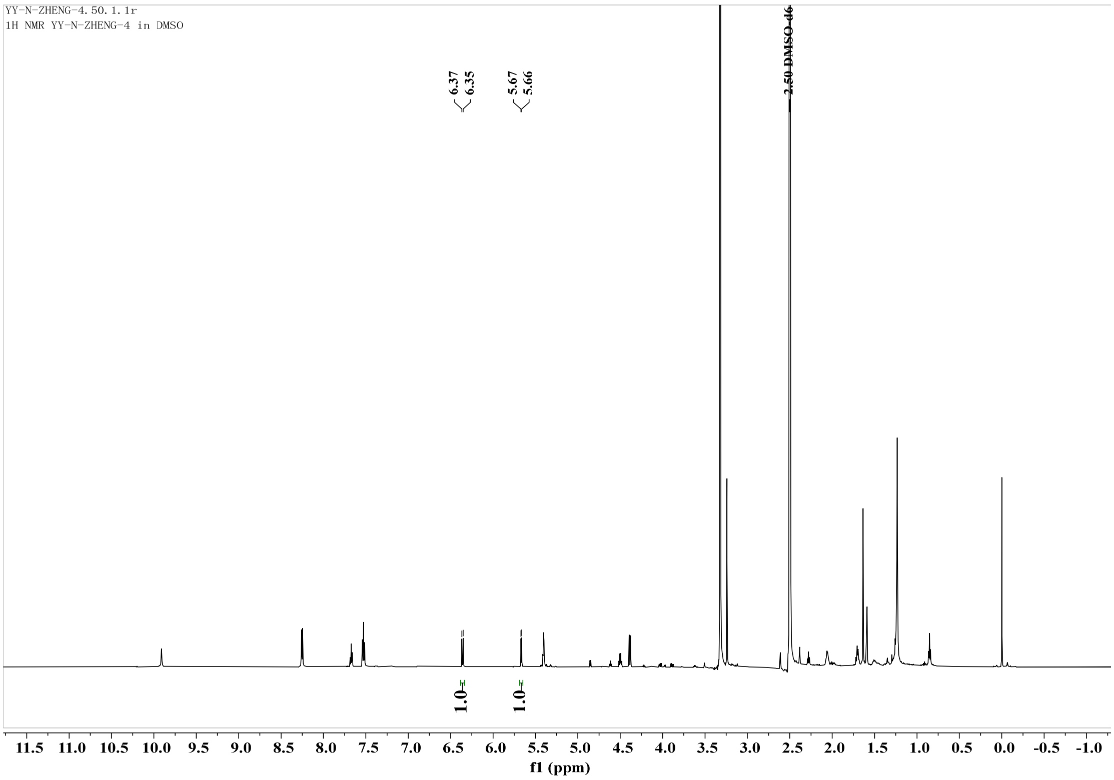

Figure S11. ^{1}H-NMR spectrum of **2** (DMSO-d_6, 600 MHz).

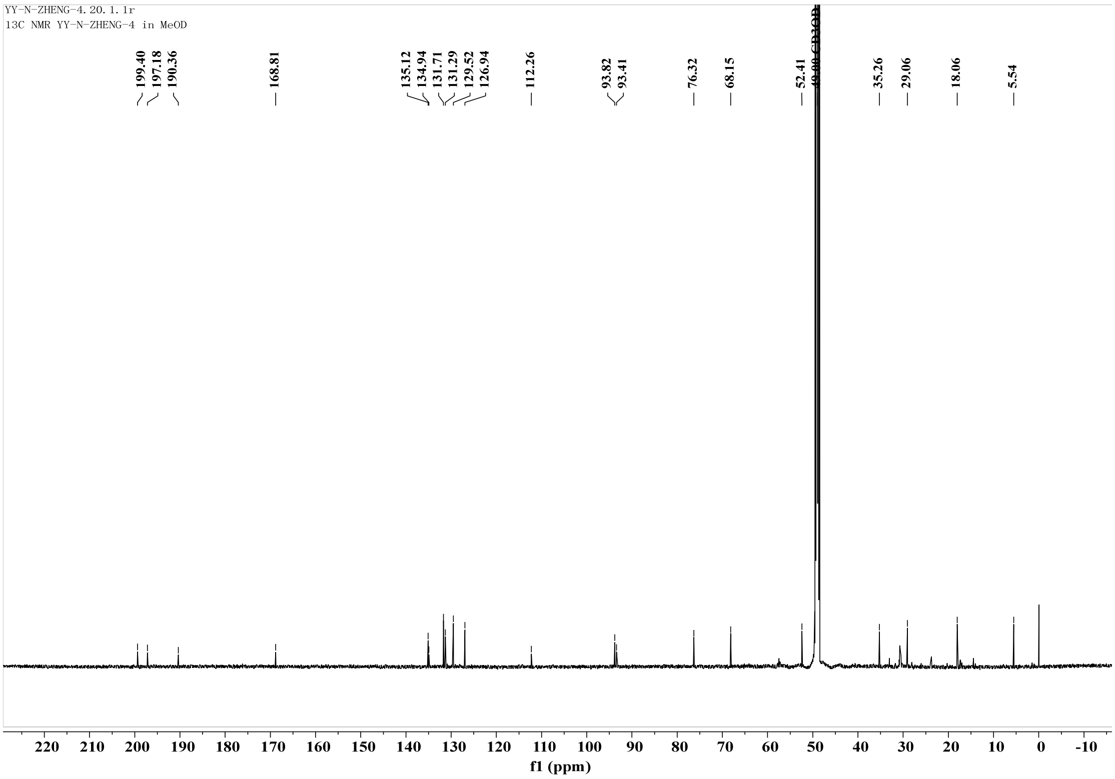

Figure S12. ^{13}C-NMR spectrum of **2** (CD_3OD, 150 MHz).

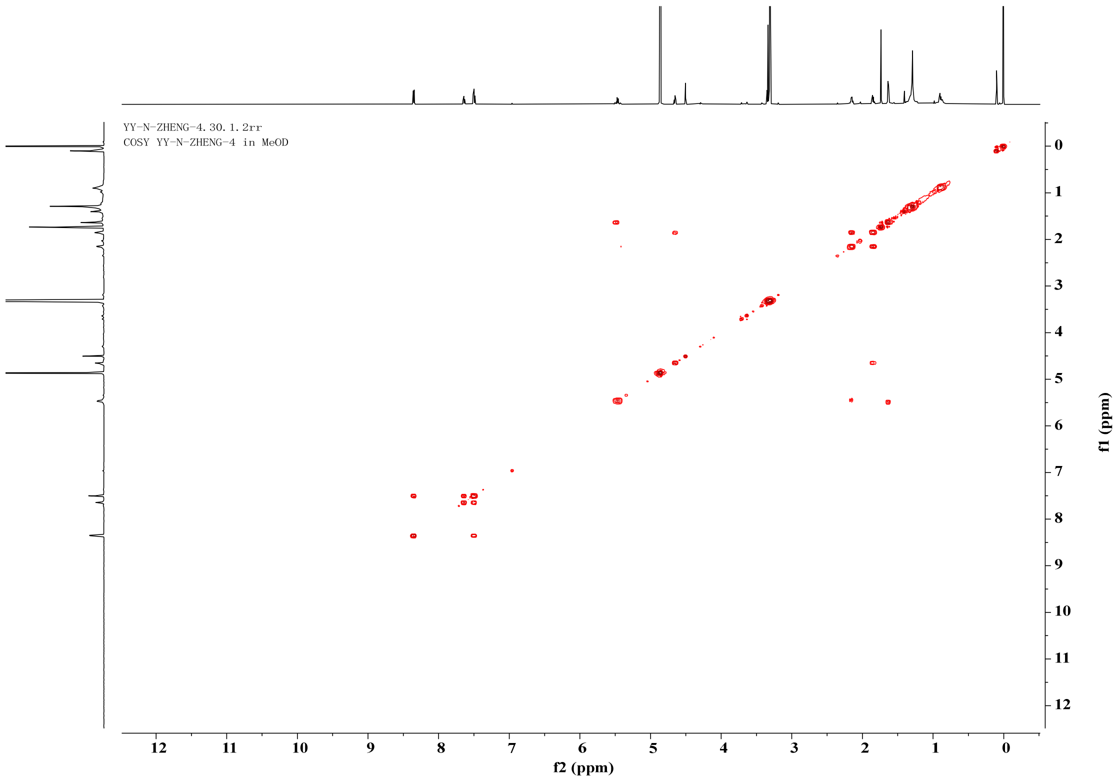

Figure S13. ^{1}H-^{1}H COSY spectrum of **2** (CD_3OD).

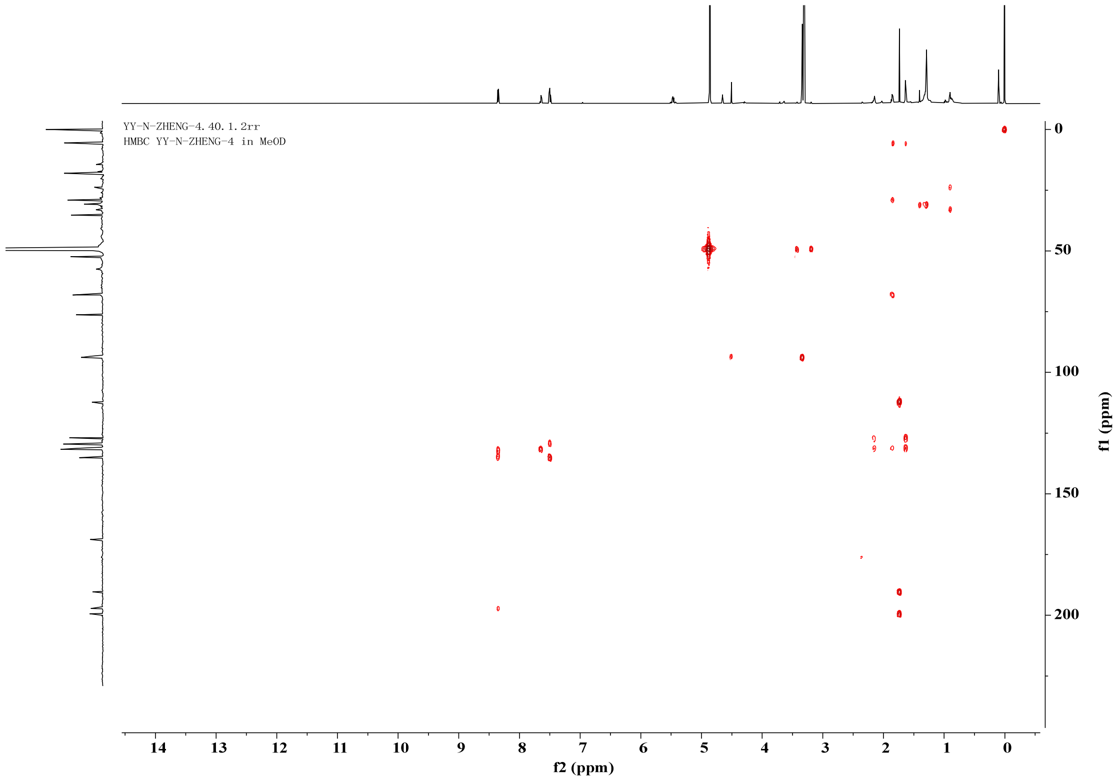

Figure S14. HMBC spectrum of **2** (CD_3OD).

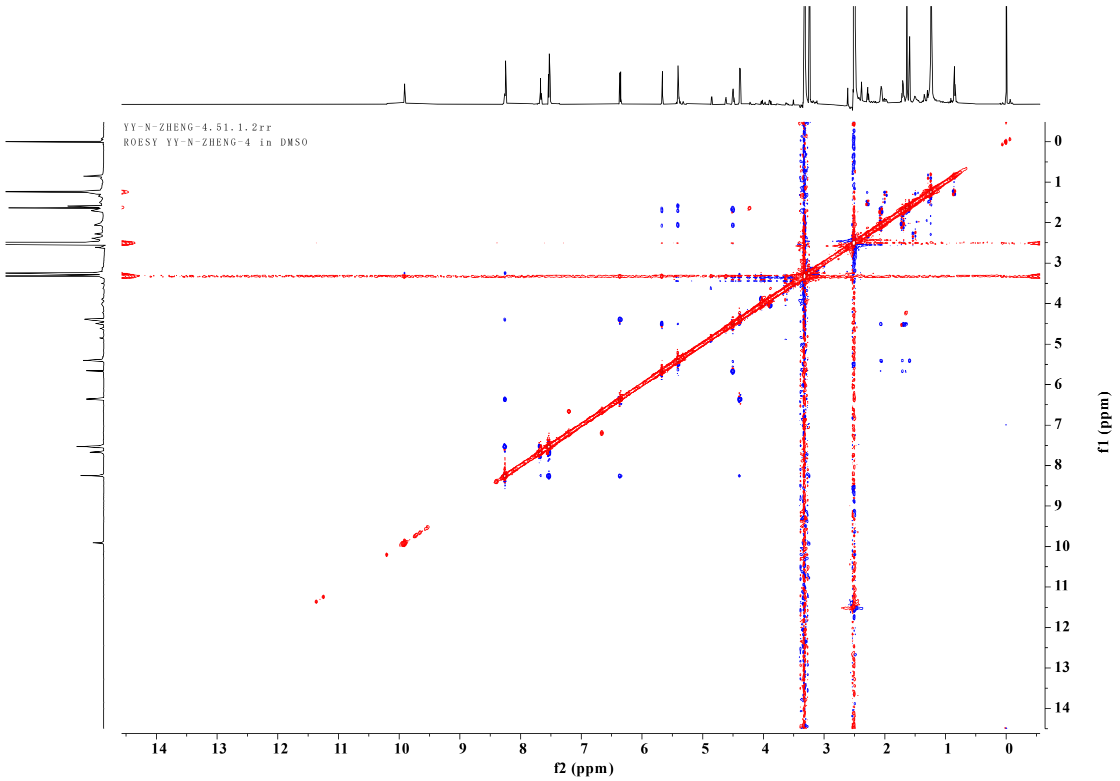

Figure S15. ROESY spectrum of **2** (DMSO-d_6).

Elemental Composition Report **Page 1**

Single Mass Analysis
Tolerance = 5.0 mDa / DBE: min = -1.5, max = 50.0
Element prediction: Off
Number of isotope peaks used for i-FIT = 3

Monoisotopic Mass, Even Electron Ions
2126 formula(e) evaluated with 1 results within limits (up to 50 best isotopic matches for each mass)
Elements Used:
C: 22-22 H: 25-25 N: 0-100 O: 0-100 Na: 0-6
240131-6-176-1-3 11 (0.076)
1: TOF MS ES+

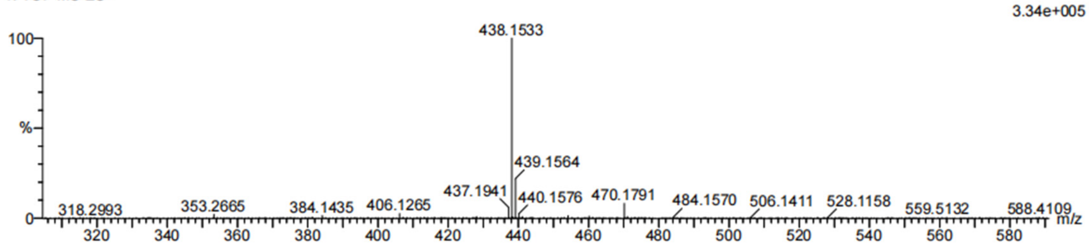

Minimum:				-1.5				
Maximum:		5.0	10.0	50.0				
Mass	Calc. Mass	mDa	PPM	DBE	i-FIT	Norm	Conf(%)	Formula
438.1533	438.1529	0.4	0.9	10.5	205.6	n/a	n/a	C22 H25 N O7 Na

Figure S16. HRESIMS spectrum of **3**.

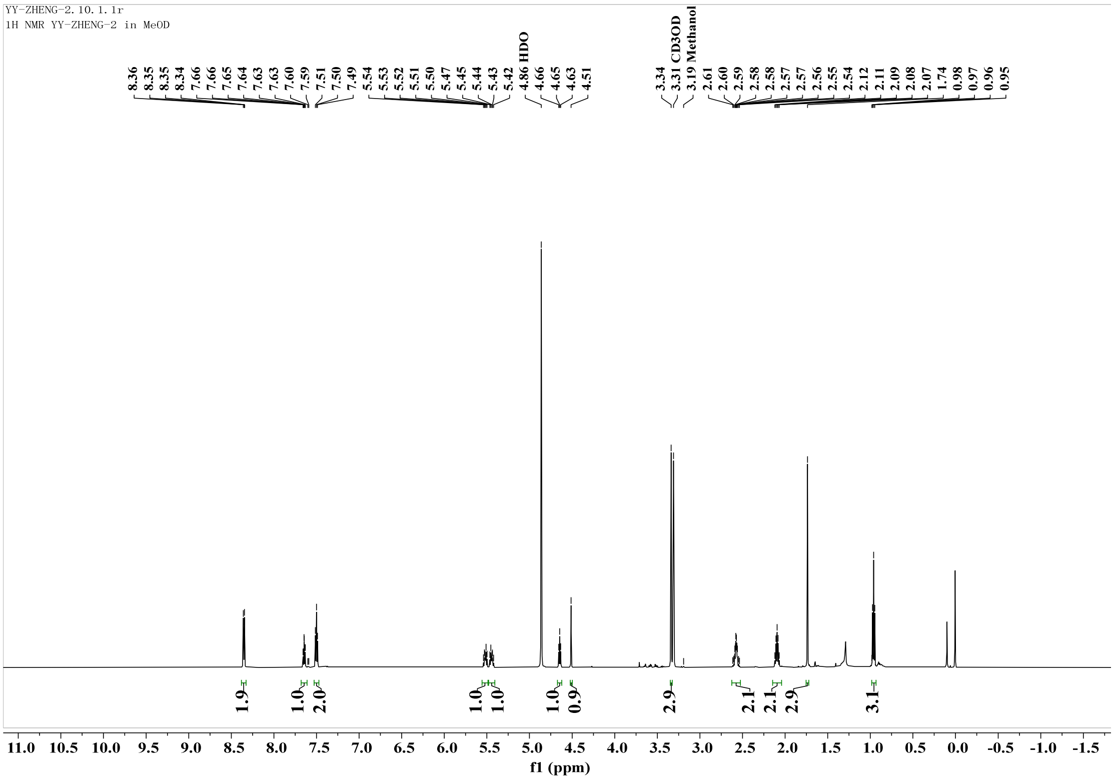

Figure S17. ^{1}H-NMR spectrum of **3** (CD_3OD, 600 MHz).

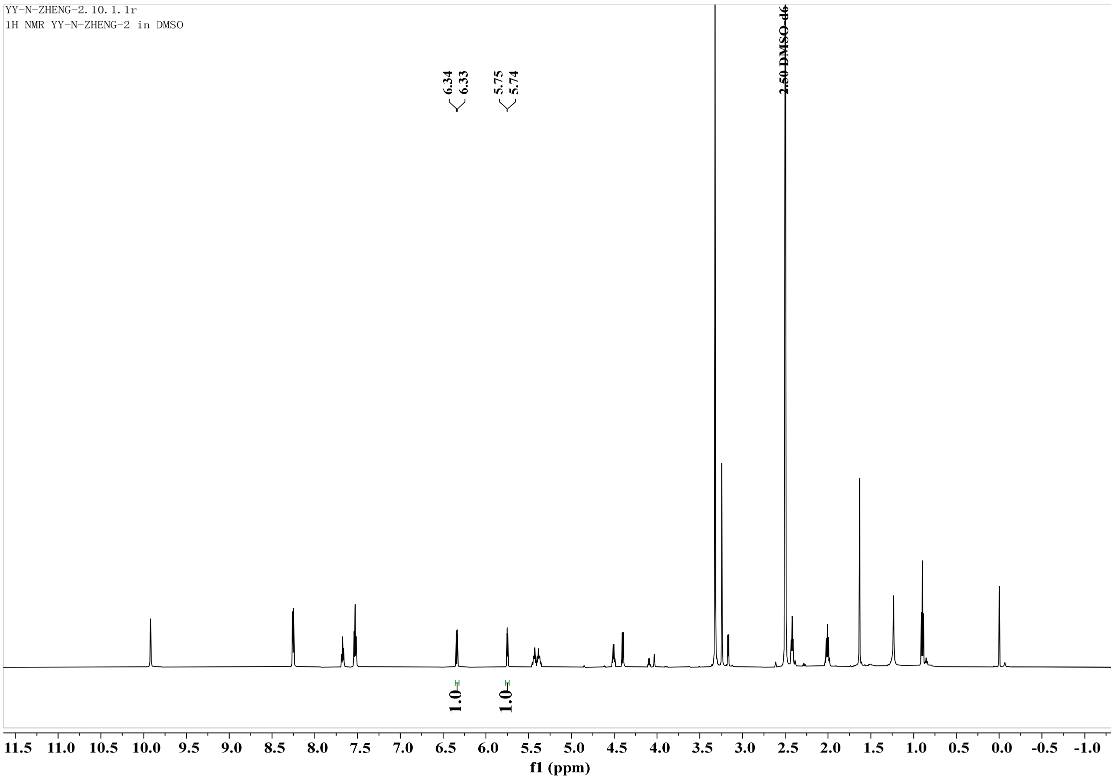

Figure S18. ^{1}H-NMR spectrum of **3** (DMSO-d_6, 600 MHz).

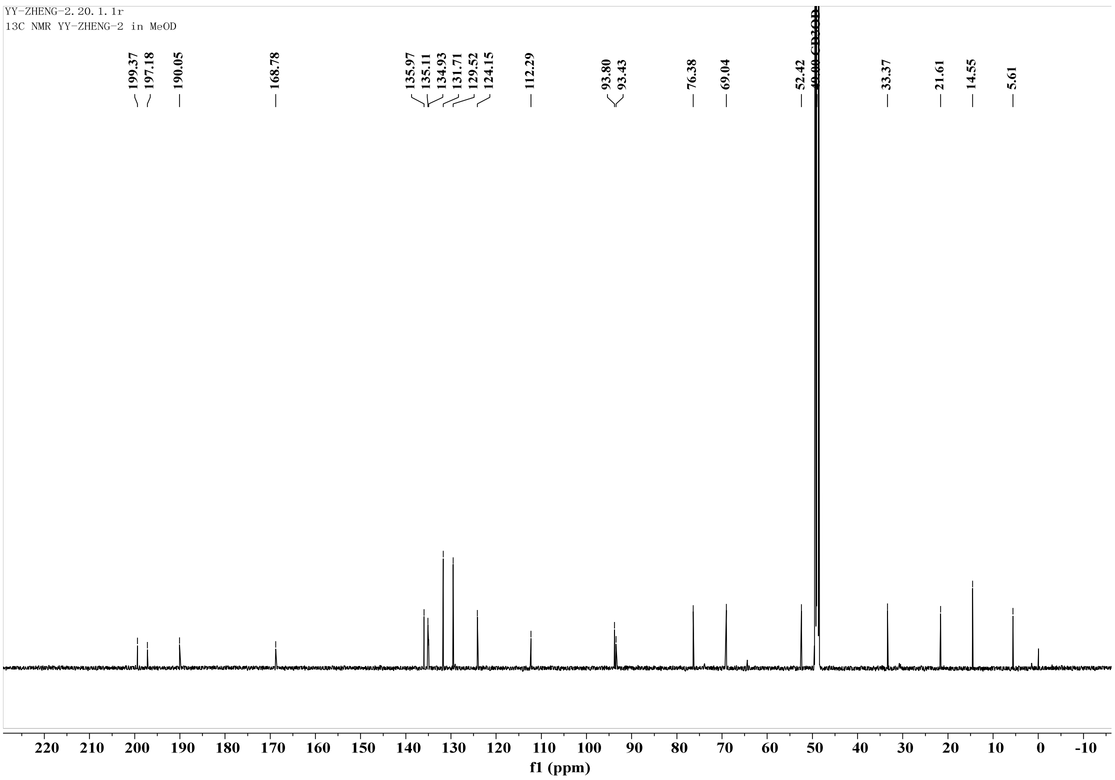

Figure S19. ^{13}C-NMR spectrum of **3** (CD_3OD, 150 MHz).

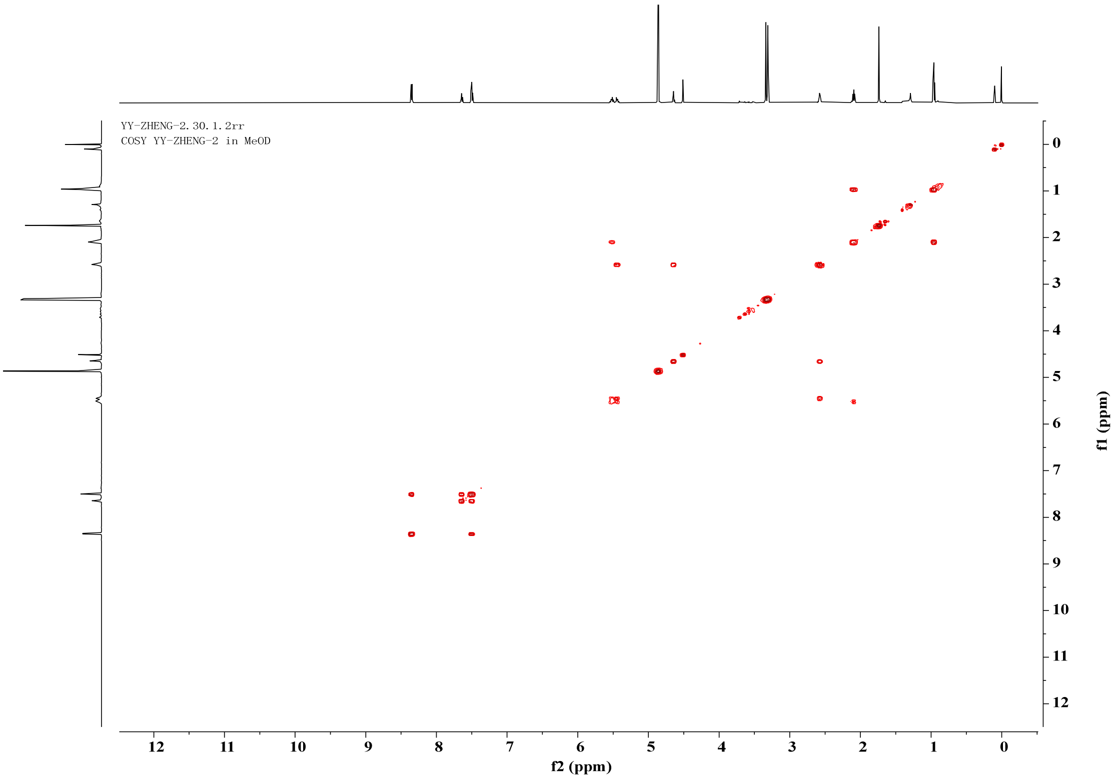

Figure S20. ^{1}H-^{1}H COSY spectrum of **3** (CD_3OD).

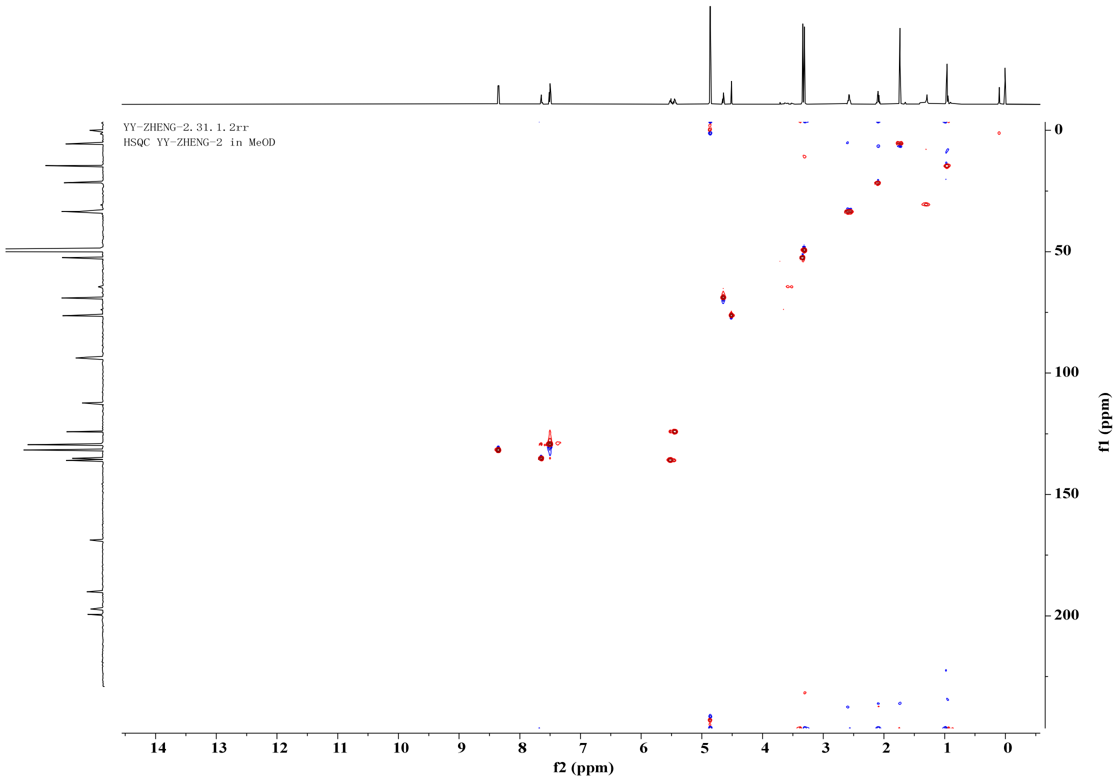

Figure S21. HSQC spectrum of **3** (CD_3OD).

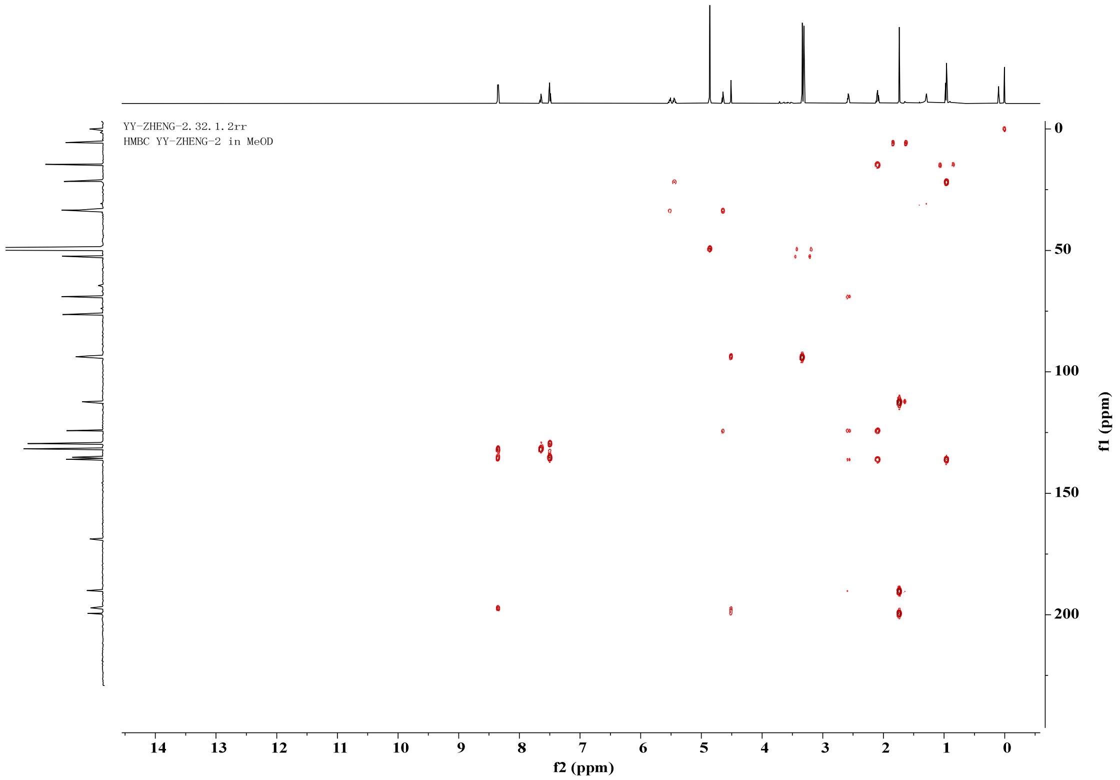

Figure S22. HMBC spectrum of **3** (CD_3OD).

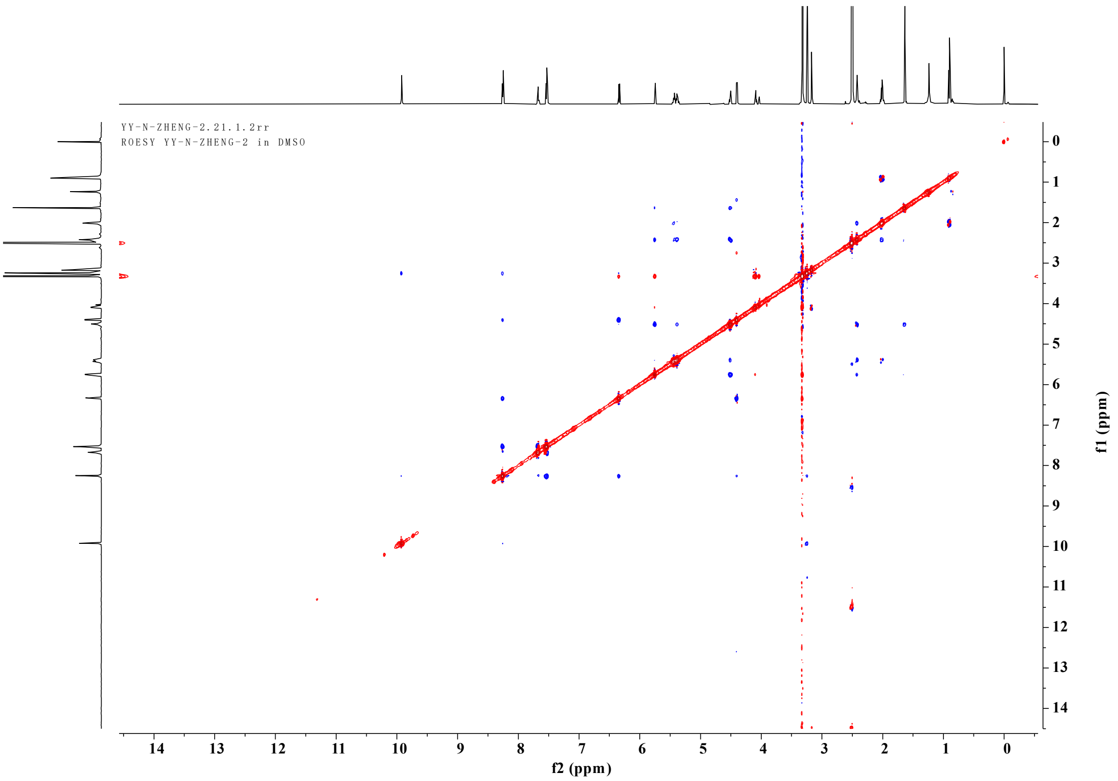

Figure S23. ROESY spectrum of **3** (DMSO-d_6).

Elemental Composition Report **Page 1**

Single Mass Analysis
Tolerance = 5.0 mDa / DBE: min = -1.5, max = 50.0
Element prediction: Off
Number of isotope peaks used for i-FIT = 3

Monoisotopic Mass, Even Electron Ions
1008 formula(e) evaluated with 1 results within limits (up to 50 best isotopic matches for each mass)
Elements Used:
C: 21-21 H: 23-23 N: 0-200 O: 0-100 Na: 0-2

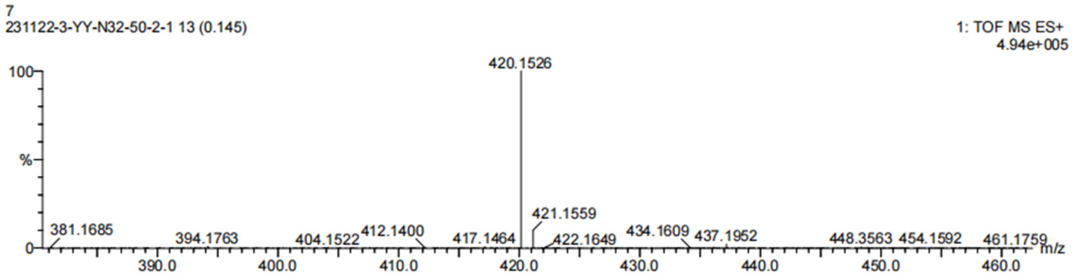

Minimum:				-1.5				
Maximum:		5.0	10.0	50.0				
Mass	Calc. Mass	mDa	PPM	DBE	i-FIT	Norm	Conf(%)	Formula
420.1526	420.1535	-0.9	-2.1	11.5	196.3	n/a	n/a	C21 H23 N3 O5 Na

Figure S24. HRESIMS spectrum of **4**.

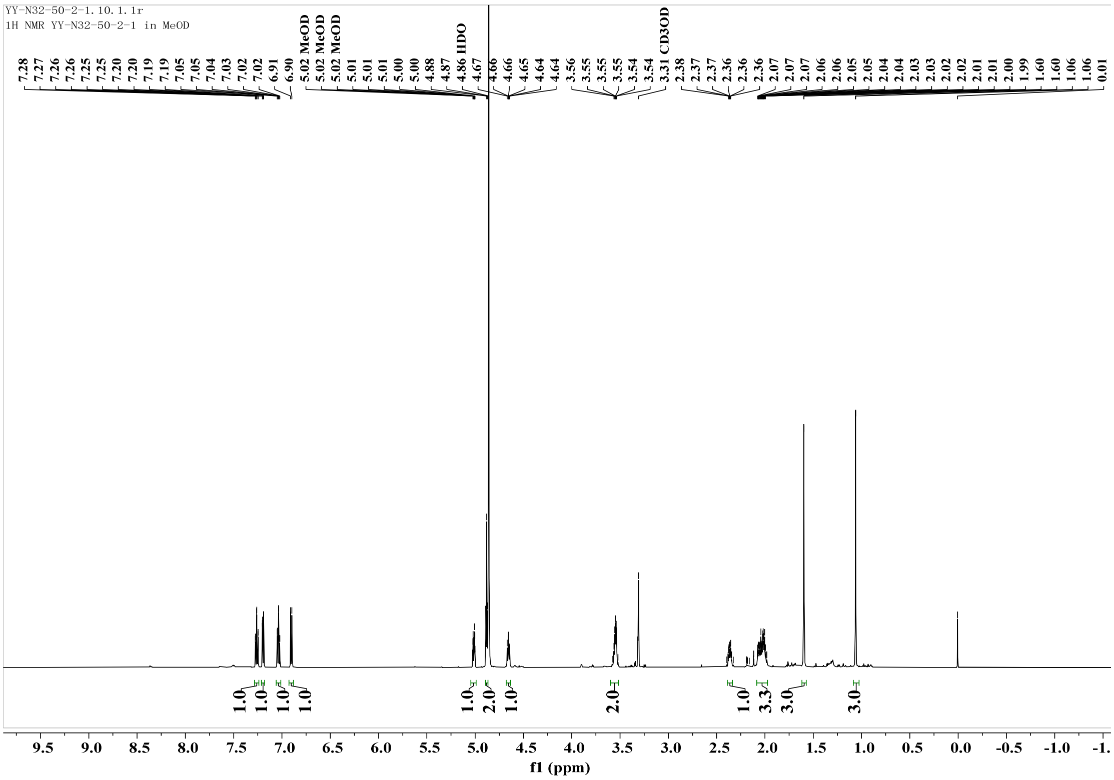

Figure S25. ^{1}H-NMR spectrum of **4** (CD_3OD, 600 MHz).

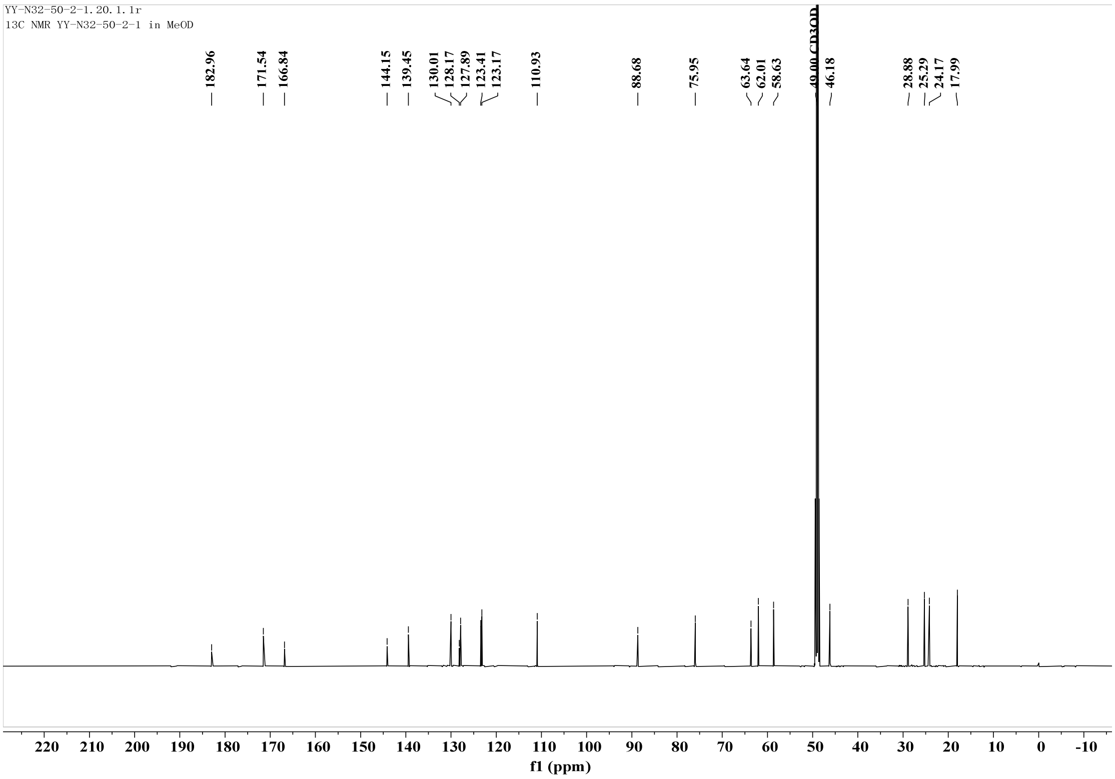

Figure S26. ^{13}C-NMR spectrum of **4** (CD_3OD, 150 MHz).

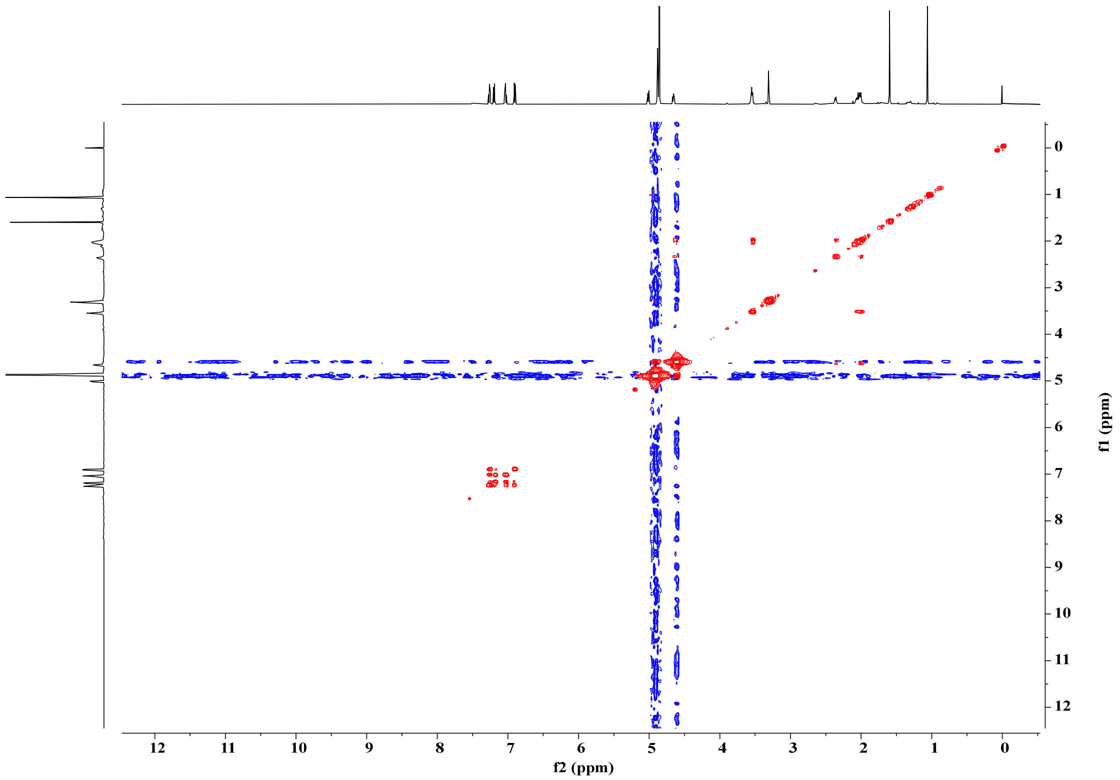

Figure S27. ^{1}H-^{1}H COSY spectrum of **4** (CD_3OD).

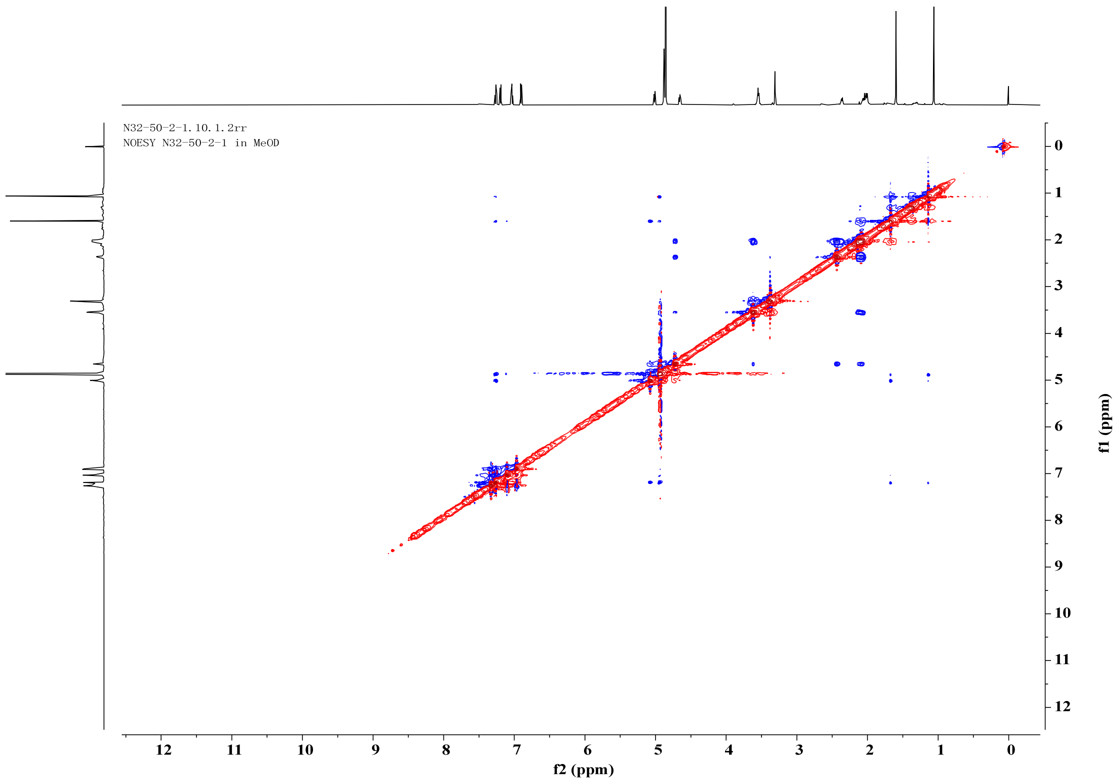

Figure S28. NOESY spectrum of **4** (CD_3OD).

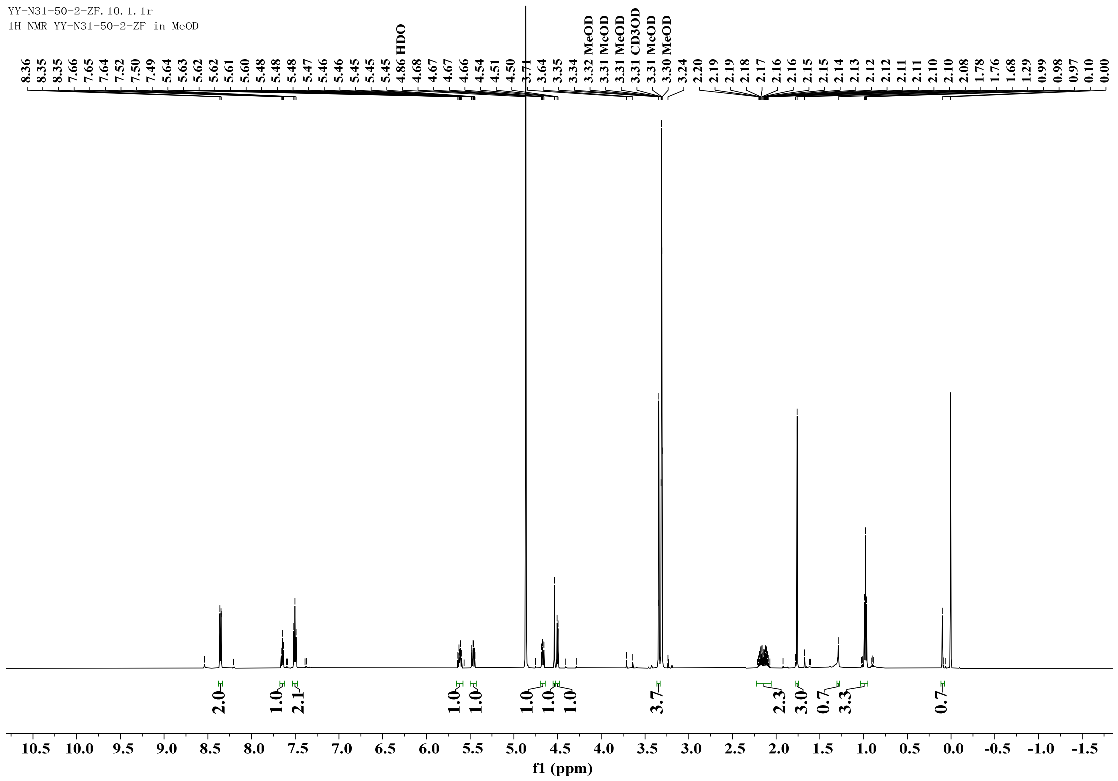

Figure S29. ^{1}H-NMR spectrum of **5** (CD_3OD, 600 MHz).

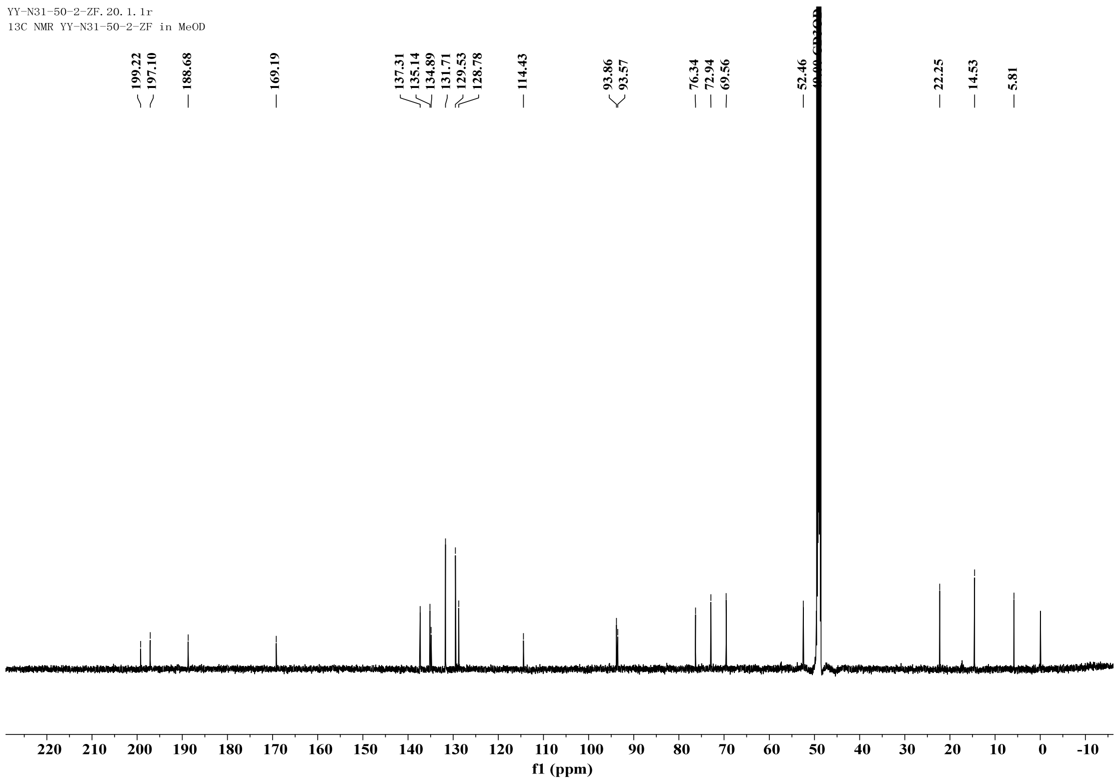

Figure S30. ^{13}C-NMR spectrum of **5** (CD_3OD, 150 MHz).

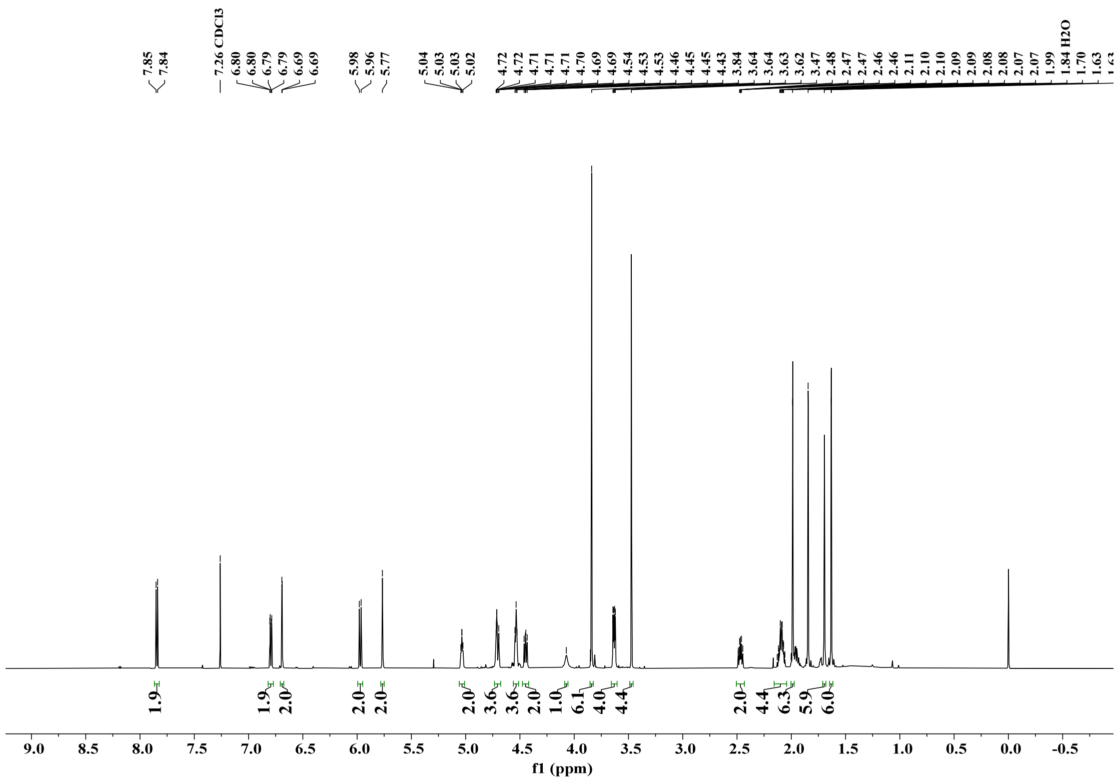

Figure S31. ^{1}H-NMR spectrum of **6** ($CDCl_3$, 600 MHz).

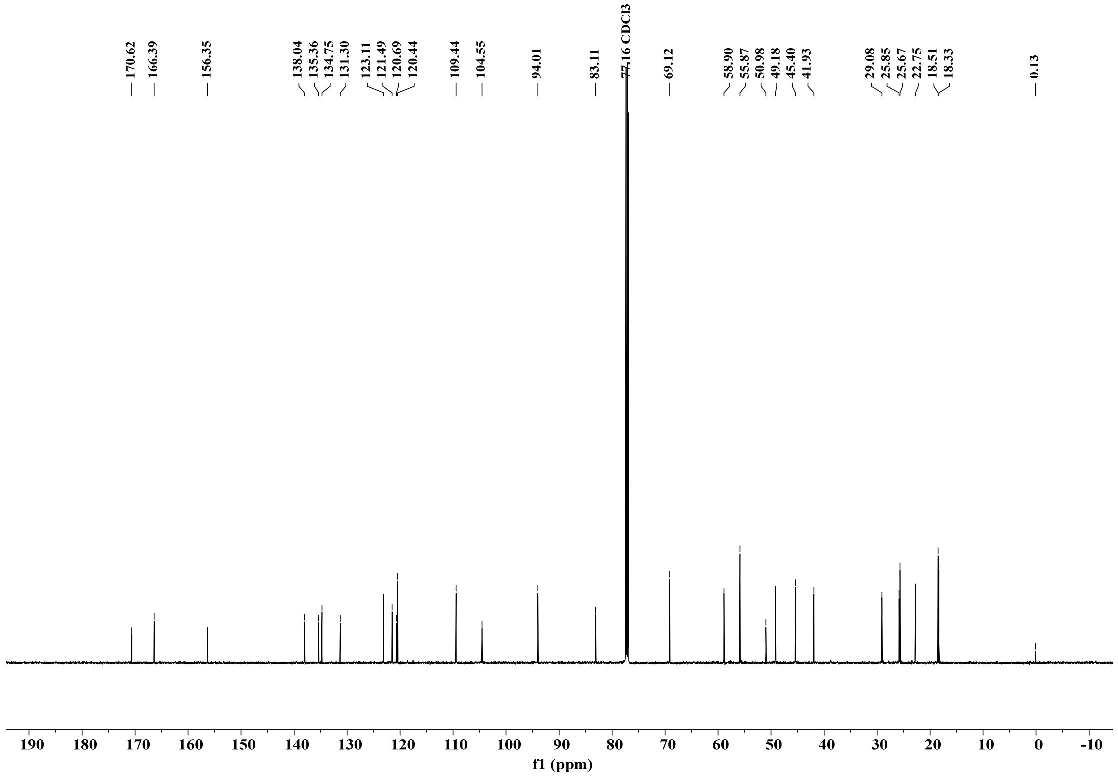

Figure S32. ^{13}C-NMR spectrum of **6** ($CDCl_3$, 150 MHz).

checkCIF/PLATON report

Structure factors have been supplied for datablock(s) FumitremorgiuB_231207

THIS REPORT IS FOR GUIDANCE ONLY. IF USED AS PART OF A REVIEW PROCEDURE FOR PUBLICATION, IT SHOULD NOT REPLACE THE EXPERTISE OF AN EXPERIENCED CRYSTALLOGRAPHIC REFEREE.

No syntax errors found.   CIF dictionary   Interpreting this report

Datablock: FumitremorgiuB_231207

```
Bond precision:   C-C = 0.0031 A           Wavelength=1.54178

Cell:              a=7.2372(4)      b=14.7130(7)      c=24.7275(12)
                   alpha=90         beta=90           gamma=90
Temperature:       153 K

                 Calculated                  Reported
Volume           2633.0(2)                   2633.0(2)
Space group      P 21 21 21                  P 21 21 21
Hall group       P 2ac 2ab                   P 2ac 2ab
Moiety formula   C27 H33 N3 O5, C H4 O       ?
Sum formula      C28 H37 N3 O6               C28 H37 C10 N3 O6
Mr               511.61                      511.60
Dx,g cm-3        1.291                       1.291
Z                4                           4
Mu (mm-1)        0.742                       0.742
F000             1096.0                      1096.0
F000'            1099.41
h,k,lmax                                     8,18,30
Nref                                         5211
Tmin,Tmax        0.862,0.935                 0.860,0.940
Tmin'            0.862

Correction method= # Reported T Limits: Tmin=0.860 Tmax=0.940
AbsCorr = MULTI-SCAN

Data completeness=                 Theta(max)= 72.640

                                                    wR2(reflections)=
R(reflections)= 0.0364( 5079)                       0.0969( 5211)

S = 1.050                  Npar= 340
```

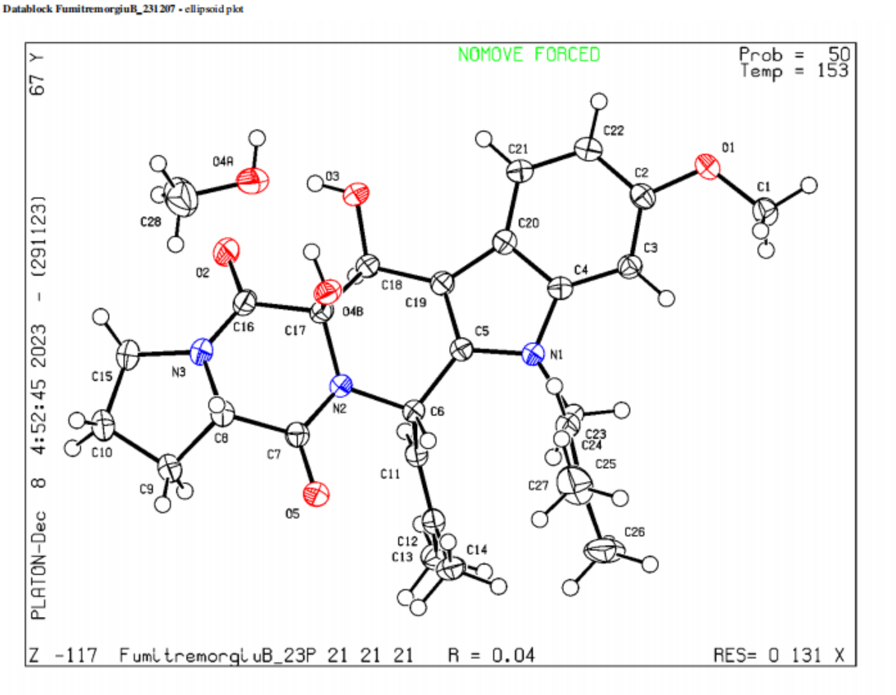

Figure S33. X-ray diffraction data of **6**.

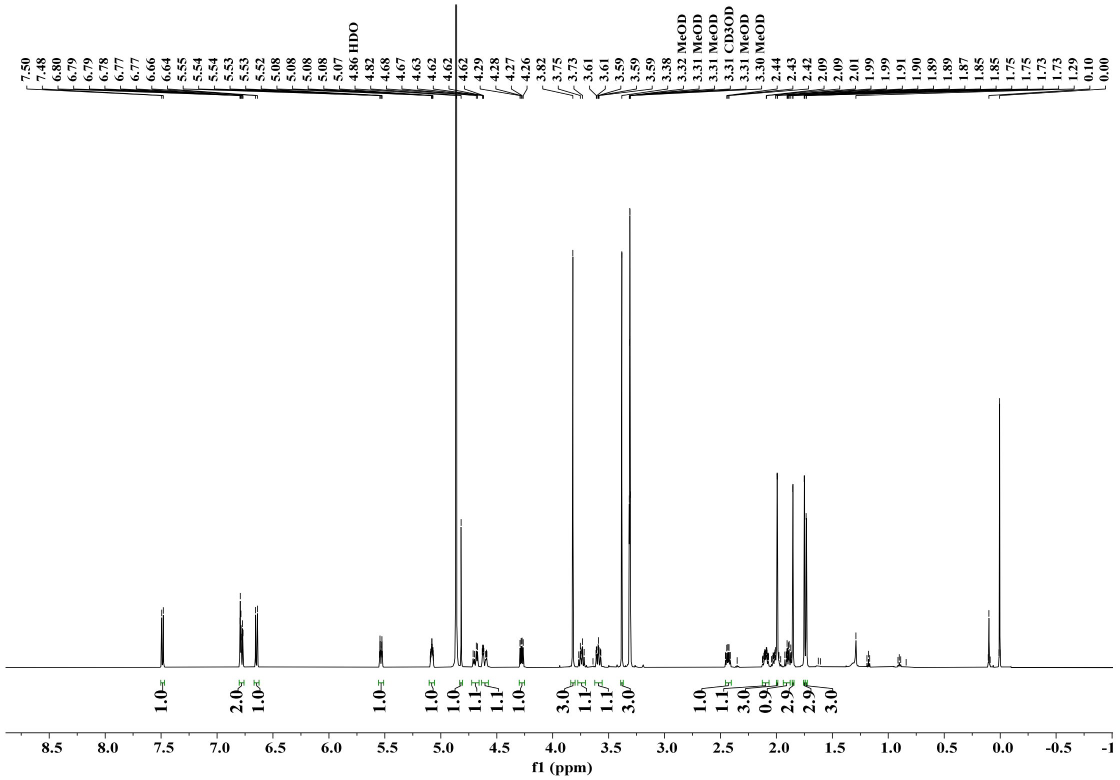

Figure S34. ^{1}H-NMR spectrum of **7** (CD_3OD, 600 MHz).

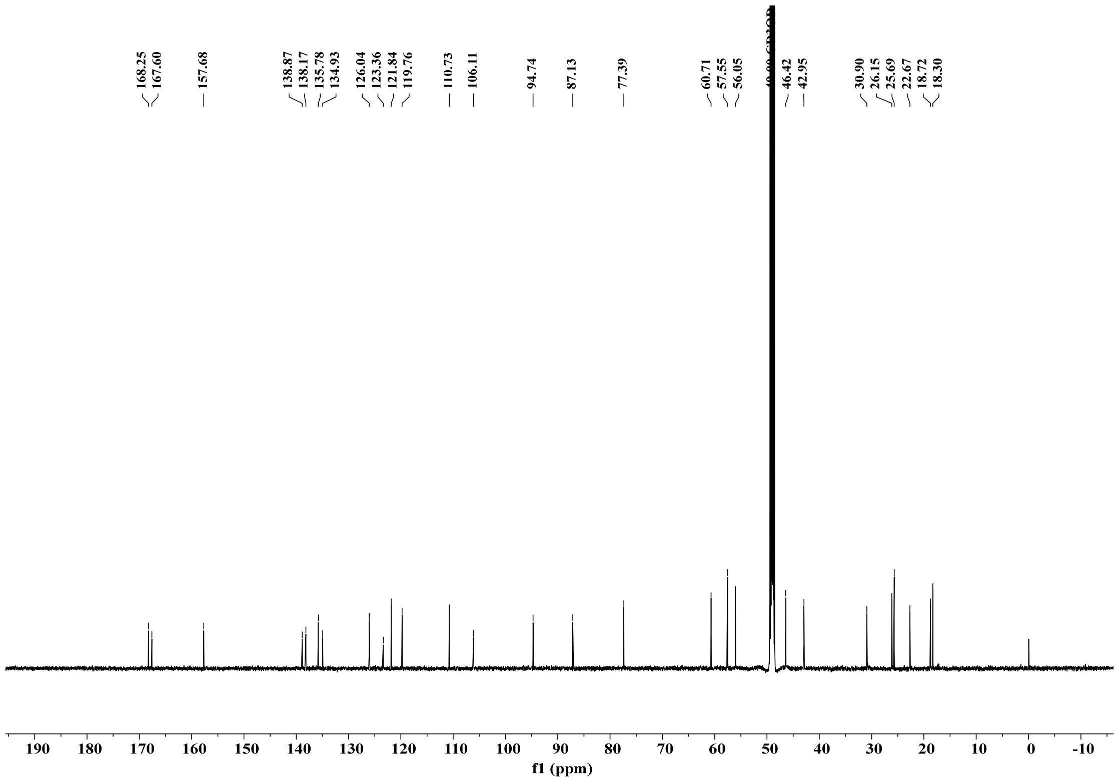

Figure S35. ^{13}C-NMR spectrum of **7** (CD_3OD, 150 MHz).

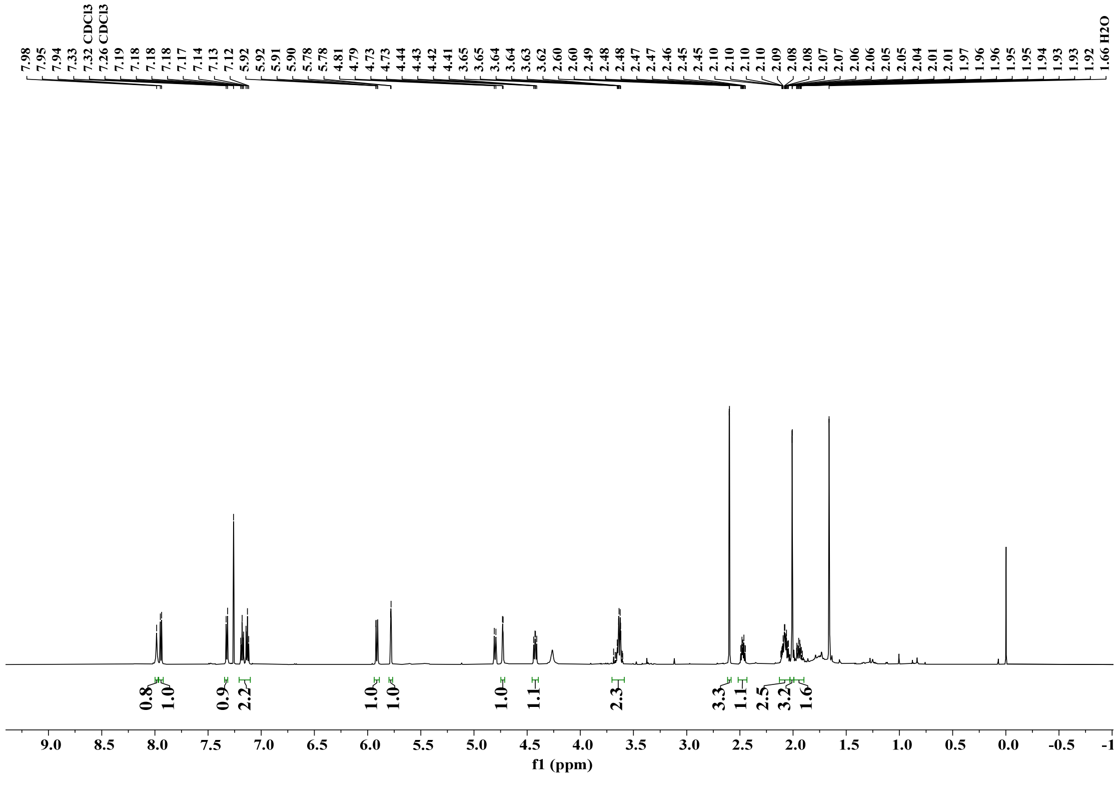

Figure S36. ^{1}H-NMR spectrum of **8** ($CDCl_3$, 600 MHz).

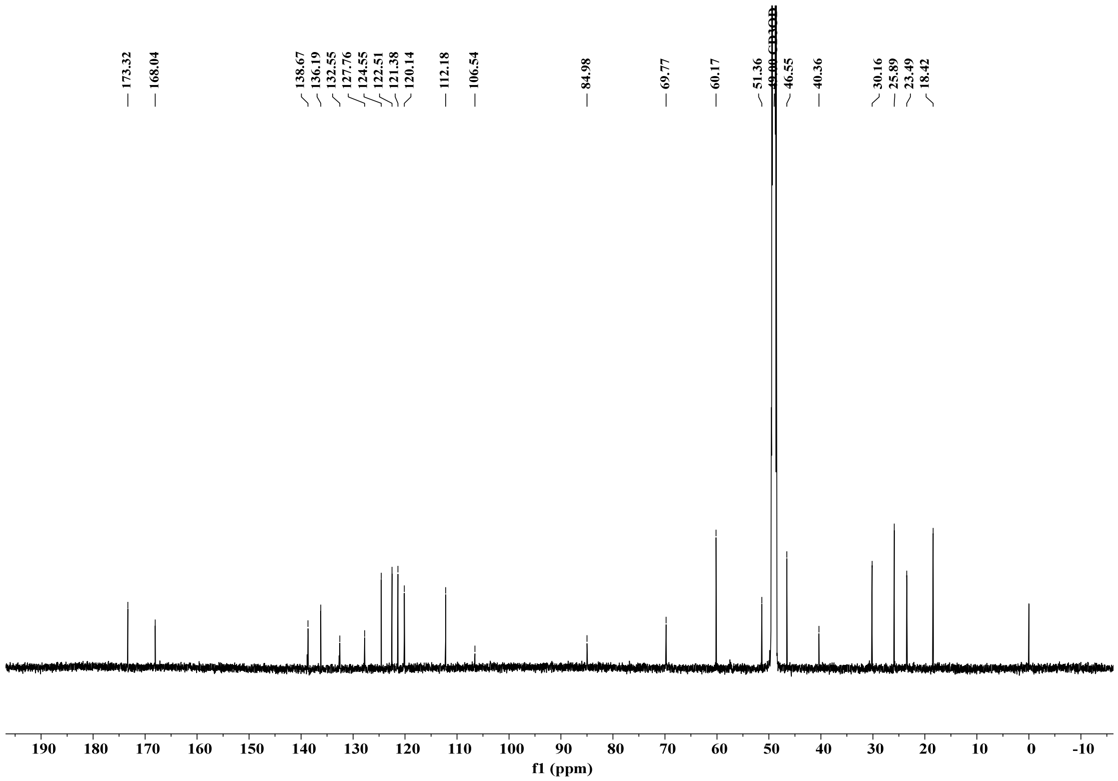

Figure S37. ^{13}C-NMR spectrum of **8** ($CDCl_3$, 150 MHz).

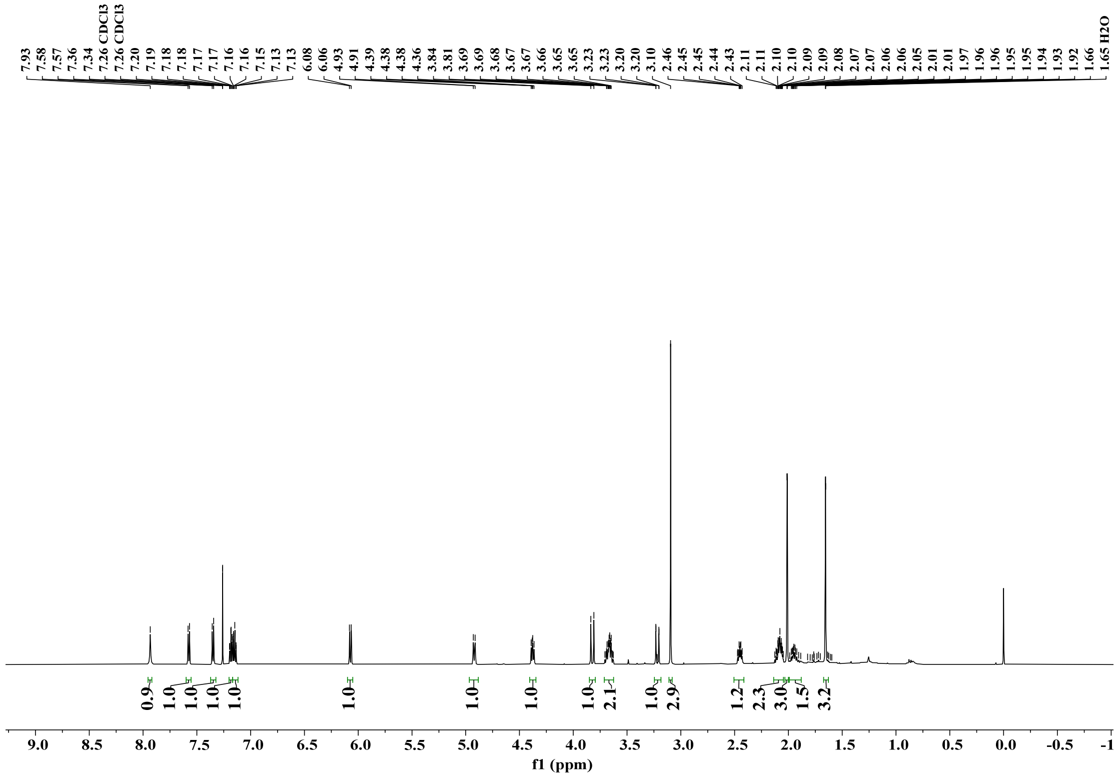

Figure S38. ^{1}H-NMR spectrum of **9** ($CDCl_3$, 600 MHz).

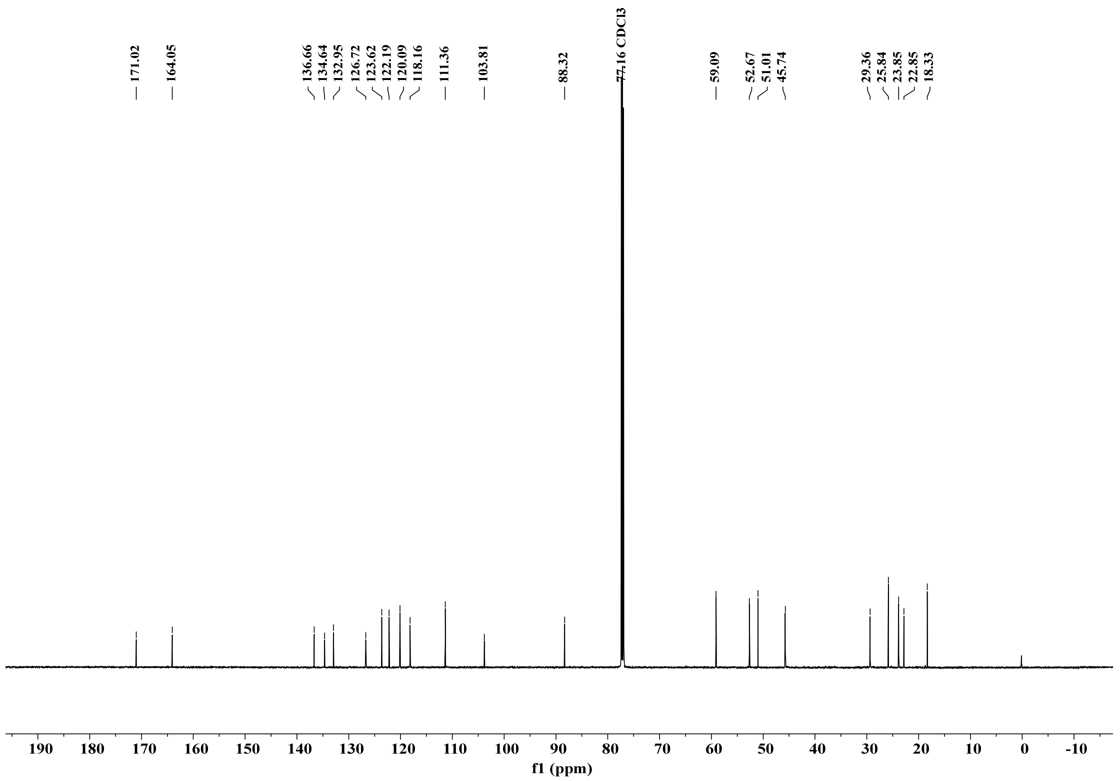

Figure S39. ^{13}C-NMR spectrum of **9** ($CDCl_3$, 150 MHz).

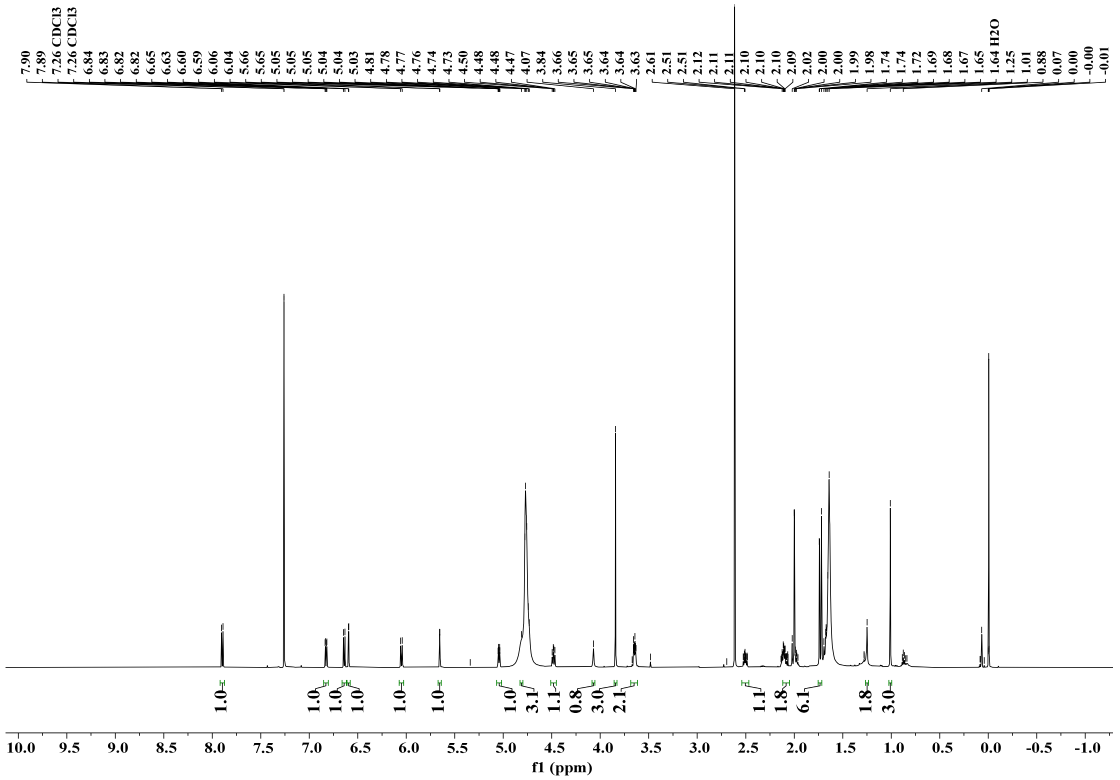

Figure S40. ^{1}H-NMR spectrum of **10** ($CDCl_3$, 600 MHz).

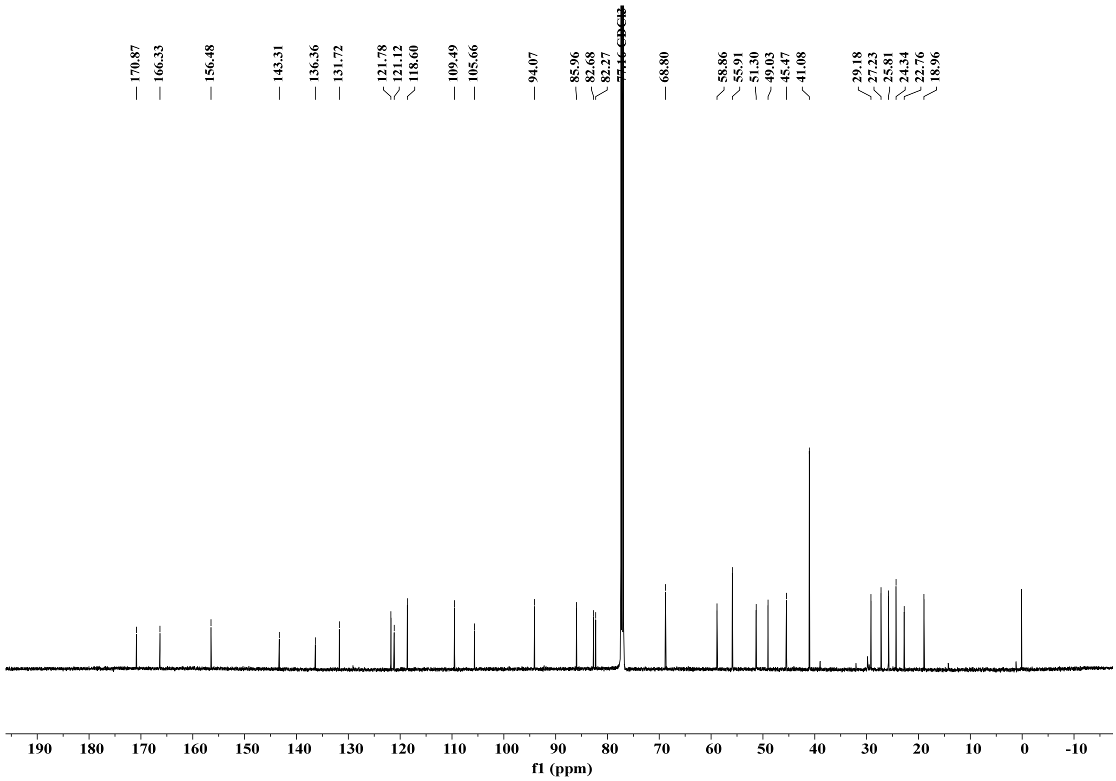

Figure S41. ^{13}C-NMR spectrum of **10** ($CDCl_3$, 150 MHz).

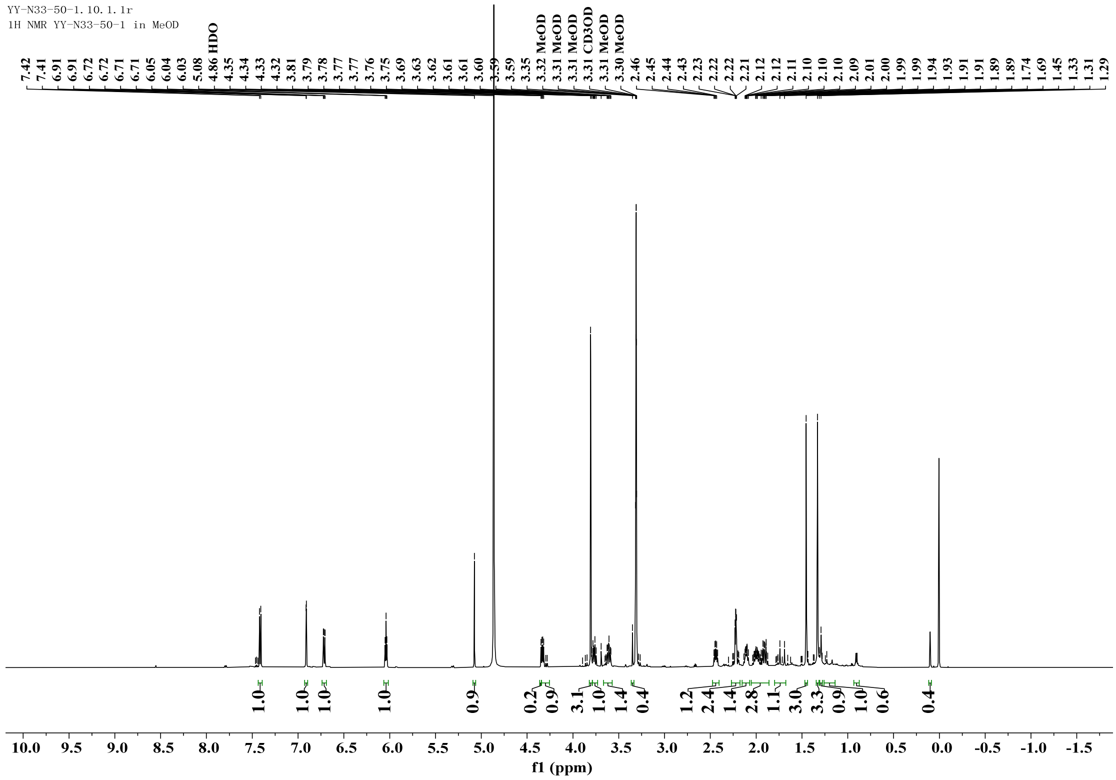

Figure S42. ^{1}H-NMR spectrum of **11** (CD_3OD, 600 MHz).

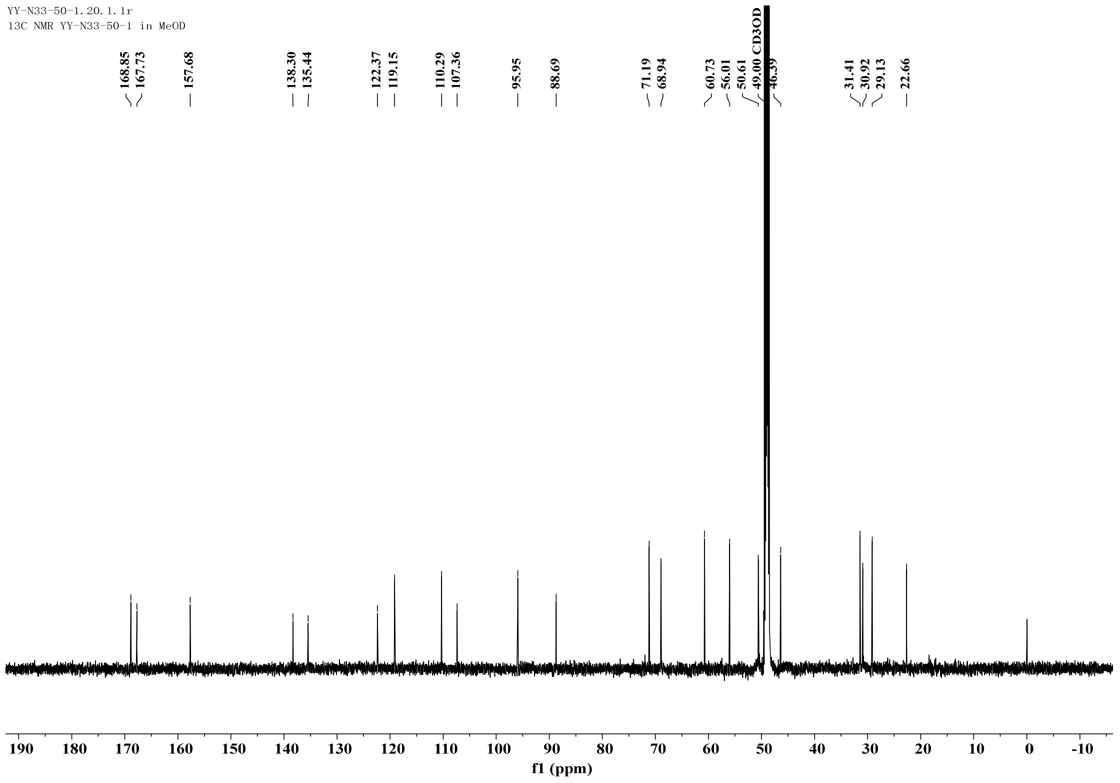

Figure S43. ^{13}C-NMR spectrum of **11** (CD_3OD, 150 MHz).

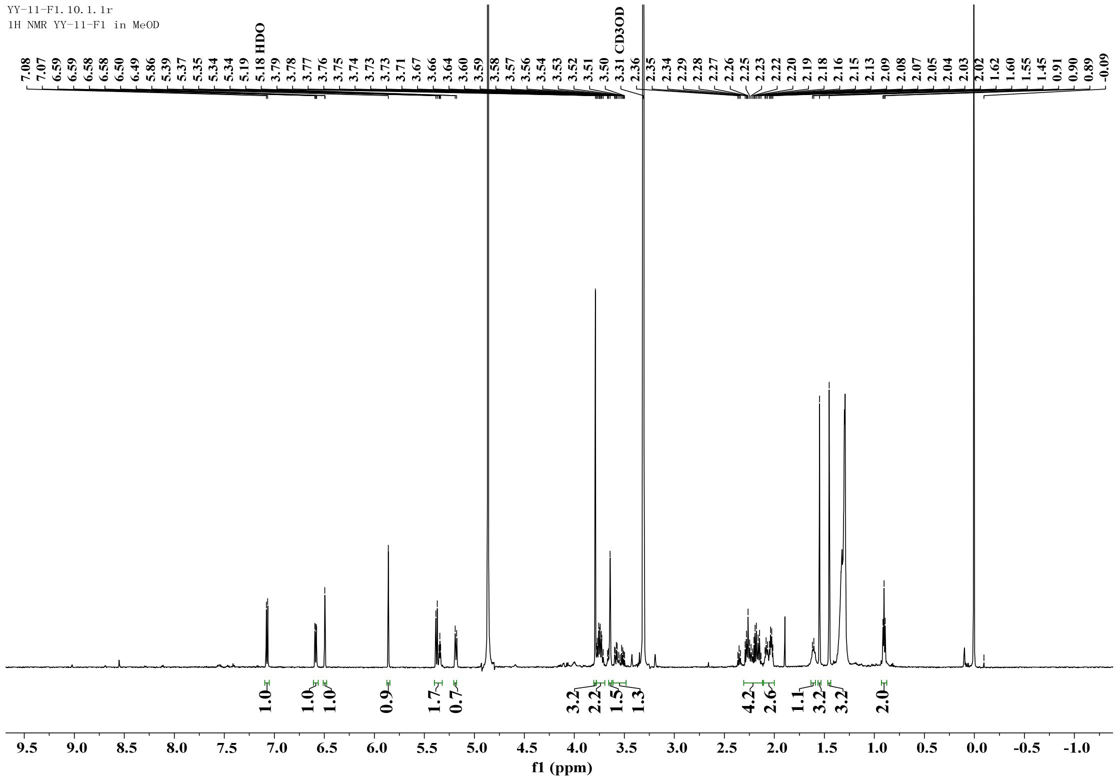

Figure S44. ^{1}H-NMR spectrum of **12** (CD_3OD, 600 MHz).

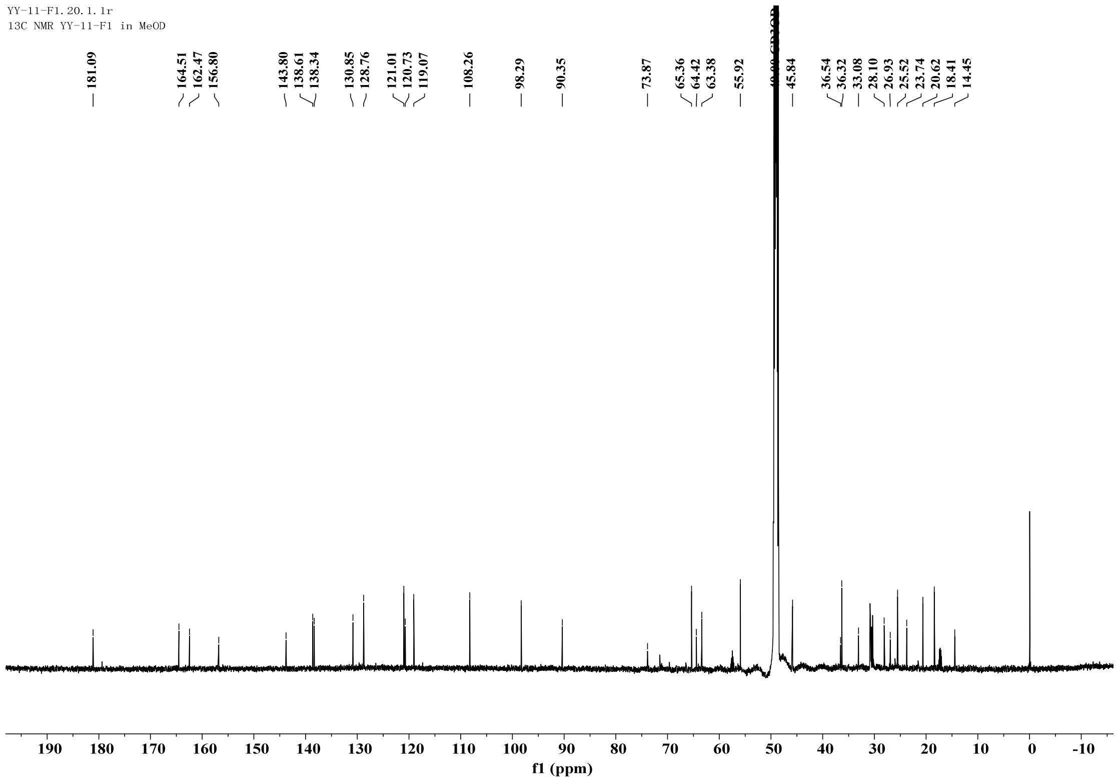

Figure S45. ^{13}C-NMR spectrum of **12** (CD_3OD, 150 MHz).
